# Manganese-Mediated Coupling Reaction of Vinylarenes and Aliphatic Alcohols

**DOI:** 10.1038/srep15250

**Published:** 2015-10-16

**Authors:** Wei Zhang, Nai-Xing Wang, Cui-Bing Bai, Yan-Jing Wang, Xing-Wang Lan, Yalan Xing, Yi-He Li, Jia-Long Wen

**Affiliations:** 1 Technical Institute of Physics and Chemistry, Chinese Academy of Sciences, Beijing, 100190, China; 2 Department of Chemistry, William Paterson University of New Jersey, 300 Pompton Road, Wayne, New Jersey 07470, United States

## Abstract

Alcohols and alkenes are the most abundant and commonly used organic building blocks in the large-scale chemical synthesis. Herein, this is the first time to report a novel and operationally simple coupling reaction of vinylarenes and aliphatic alcohols catalyzed by manganese in the presence of TBHP (*tert*-butyl hydroperoxide). This coupling reaction provides the oxyalkylated products of vinylarenes with good regioselectivity and accomplishes with the principles of step-economies. A possible reaction mechanism has also been proposed.

Advance of organic chemistry has driven extensive research toward the development of energy-efficient catalytic methods to afford desired products from cheap, readily available and environmentally benign synthetic building blocks. Transition-metal-catalyzed reactions are versatile methods for C-C bond formations and have been widely applied in organic synthesis^1^,^2^,^3^,^4^,^5^,^6^,^7^,^8^,^9^. In particular, transition-metal-catalyzed functionalization of C-H bonds has attracted considerable interest of organic chemists because it offers efficient ways for the construction of complex molecules^9^,^10^,^11^,^12^,^13^,^14^,^15^,^16^,^17^,^18^. Direct activation and functionalization of C-H bonds are ideal and environmentally attractive strategies because stoichiometric amounts of halogenated or organometallic byproducts are not generated. Vinylarenes are common intermediates^19^,^20^, and their difunctionalization to form C-X and C-C bonds is an important method in organic synthesis for the construction of complex molecules^21^,^22^,^23^,^24^,^25^.

Although there has been remarkable progress in the transition-metal-catalyzed difunctionalization of vinylarenes in the past decades, including diamination^26^,^27^,^28^,^29^,^30^,^31^,^32^,^33^,^34^, dioxygenation^35^,^36^,^37^,^38^,^39^, and oxyamidation^40^,^41^,^42^,^43^, there is no report of difunctionalization reaction of vinylarenes with a series of aliphatic alcohols to give the oxyalkylated products of vinylarenes (Fig. 1C). Recently, some C-H bond functionalization reactions involving vinylarenes or aliphatic alcohols have been reported. For example, in 2009, Zhang’s group reported the difunctionalization reaction of vinylarenes with cyclic ethers using copper catalysts via direct activation of *α*-sp^3^ C-H bonds of oxygen atom in the presence of *tert*-butyl hydroperoxide (TBHP) (Fig. 1A)^22^, and Liu’s group disclosed an efficient method to prepare allylic alcohols via direct C-C bond formation using alkynes and aliphatic alcohols by TBHP^44^. In 2011, Yi’s group described a selective catalytic alkylation reaction of alkenes with alcohols that forms a C-C bond between vinyl carbon-hydrogen and carbon-hydroxy centers with the concomitant loss of water (Fig. 1B)^45^. Alcohol is a highly attractive class of alkylating reagent because they are inexpensive and often easily derived from natural sources. Highly efficient and creative construction of complex molecules by one-pot reactions using cheap and readily available materials has been a challenging research in synthetic organic chemistry. Here, we reported a novel and operationally simple transition-metal-catalyzed difunctionalization reaction of vinylarenes with aliphatic alcohols, wherein MnCl_2_·4H_2_O catalyzed the regioselective oxyalkylation of vinylarenes through the cleavage of a sp^3^ C-H bond with a hydroxyl group to give the aryl products bearing *α*-carbonyl and *β*-alkyl in the presence of TBHP under mild conditions. To the best of our knowledge, this is an unprecedented difunctionalization reaction of simple vinylarenes with aliphatic alcohols to obtain complex products.

## Results

Palladium, nickel and copper and other transition metals have been widely studied in functionalization of C-H bonds because of their promise as highly efficient catalysts, but rare examples of Mn-catalyzed C-H activation reactions have been reported^46^,^47^,^48^,^49^,^50^. The effect of this non-expensive and readily available catalyst in difunctionalization of vinylarenes is still unclear. Initially, in the exploration of new metal-catalyzed reactions by C-H bond activation, we investigated the difunctionalization reaction of styrene and methanol using MnCl_2_·4H_2_O as the catalyst in the presence of 3 equiv of aqueous TBHP. It was found that the reaction afforded oxyalkylation product 3-hydroxy-1-phenylpropan-1-one (**3a**) in 65% yield (Table 1, entry 1). Styrene did not react with methanol without the catalyst or additive oxidant (Table 1, entries 2 and 3), therefore, the presence of both MnCl_2_·4H_2_O and TBHP was essential for this reaction. Reducing the amount of TBHP and enhancing or reducing the reaction temperature resulted in decrease of the yield (Table 1, entries 4, 9 and 10). Increasing the amount of aqueous TBHP or using anhydrous TBHP did not improve the yield of compound **3a,** only 62% and 64%, respectively (Table 1, entries 5 and 25). When different oxidizing agents were used, for example, NBS, DDQ, BPO, DTBP and DCP, the reaction failed to give the product **3a** (Table 1, entries 6-8, 24 and 26). Increasing the amount of MnCl_2_·4H_2_O led to a decrease of the yield of product **3a** (Table 1, entry 11). The other transition metal catalysts were either less efficient (NiCl_2_·6H_2_O, ZnCl_2_, Ni(OAc)_2_·4H_2_O, Mn(OAc)_2_·4H_2_O, CrCl_3_·6H_2_O) or incompetent (Co(OAc)_2_·4H_2_O, CoCl_2_·6H_2_O, CuBr_2_, FeCl_3_·6H_2_O, Zn(OAc)_2_·2H_2_O, CuCl_2_, CuI) (Table 1, entries 12-23).

**Figure 2:**
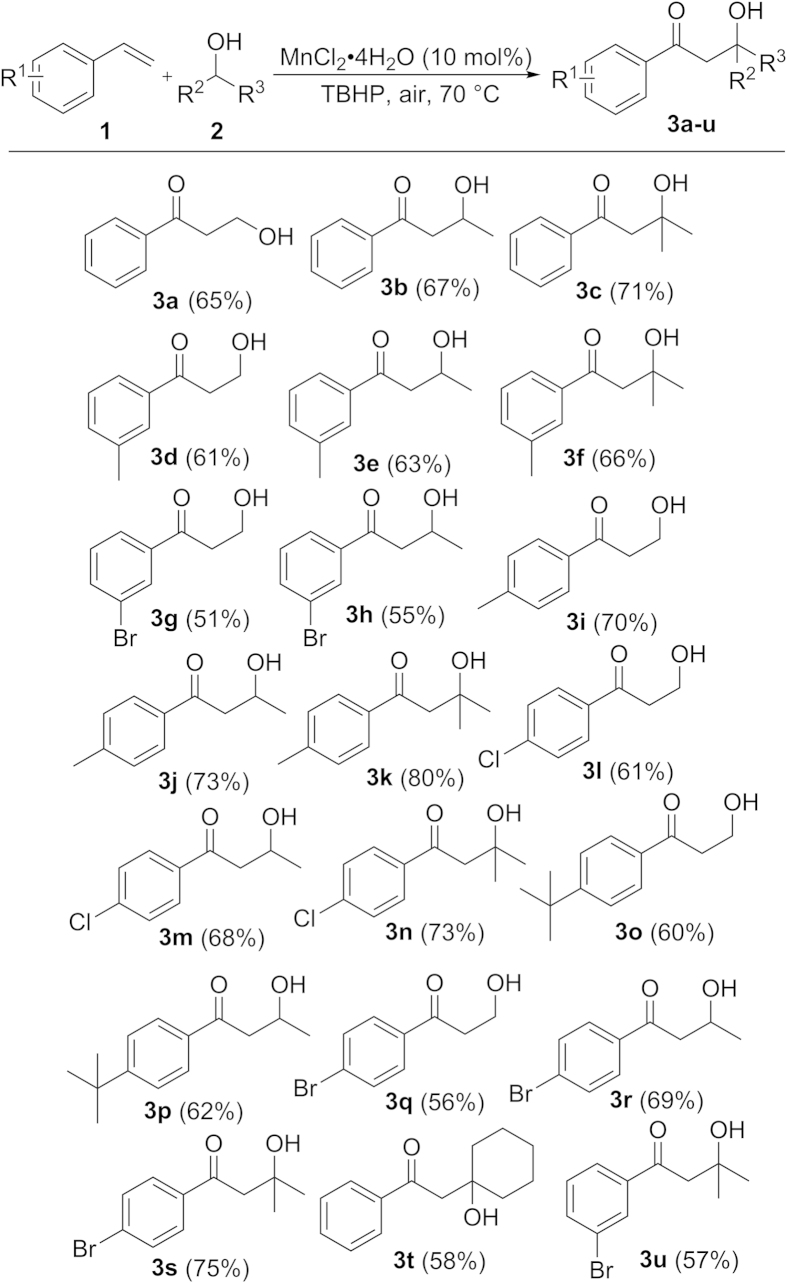
Mn-catalyzed difunctionalization reactions of vinylarenes with aliphatic alcohols under the optimized conditions.

With the optimized reaction conditions in hand, we tested the scope of this manganese-catalyzed oxyalkylation reaction. A variety of vinylarenes and aliphatic alcohols were used as substrates. The related products were showed in Fig. 2 with moderate to good yields. In general, both electron-rich and electron-deficient vinylarenes reacted easily with aliphatic 1° or 2° alcohols (Fig. 2). The yields were slightly lower when substitutions were at the 3-position of vinylarenes (Fig. 2, 3d,i,g,q). It’s noteworthy that substituted vinylarenes with electron donating group gave better yields (Fig. 2, 3f,j,k). Besides chain alcohols, cyclohexanol could also be used as a reactant and the desired product was obtained (Fig. 2, 3t). But when we used *tert*-butyl alcohol as the reaction substrate under the same conditions, no desired oxyalkylation product was obtained. This phenomenon also explained that the carbon-hydrogen bonds with hydroxyl groups were involved in difunctionalization reaction. We observed excellent regioselectivities from this difunctionalization reaction of vinylarenes. The addition of the carbon-hydrogen bonds with hydroxyl groups to vinylarenes gave the alkylated products, and then the oxidative carbonylation occurred at the *α*-position of vinylarenes.

**Figure 3:**
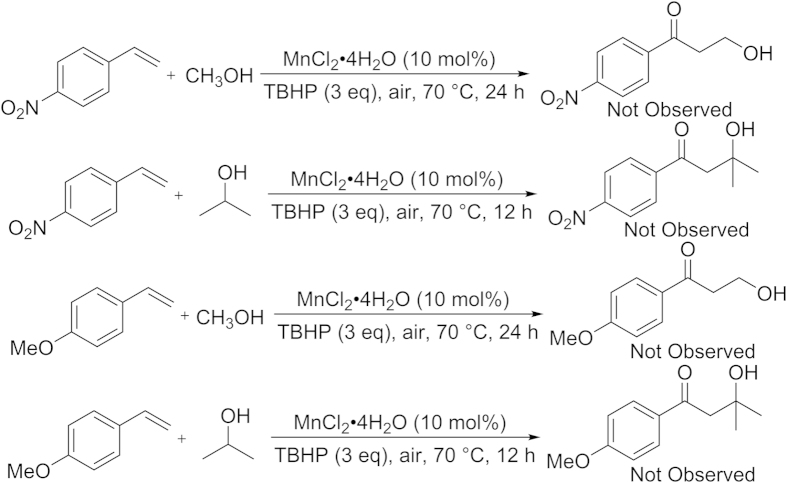
Reactions of vinylarenes with alcohols.

But it is surprising that when 4-nitrostyrene or 4-methoxystyrene was employed as reaction substrate, no oxyalkylation products were obtained and a series of the undesired compounds were generated in the process (Fig. 3). Perhaps it is because the electronic properties of the substituents affect the stability of free-radical intermediates^21^,^22^,^51^,^52^. When *α*-methylstyrene reacted with methanol, it did not provide the oxyalkylated products of vinylarenes, which was expected. When *trans*-stilbene reacted with methanol, it only gave the trace target product.

**Figure 4:**
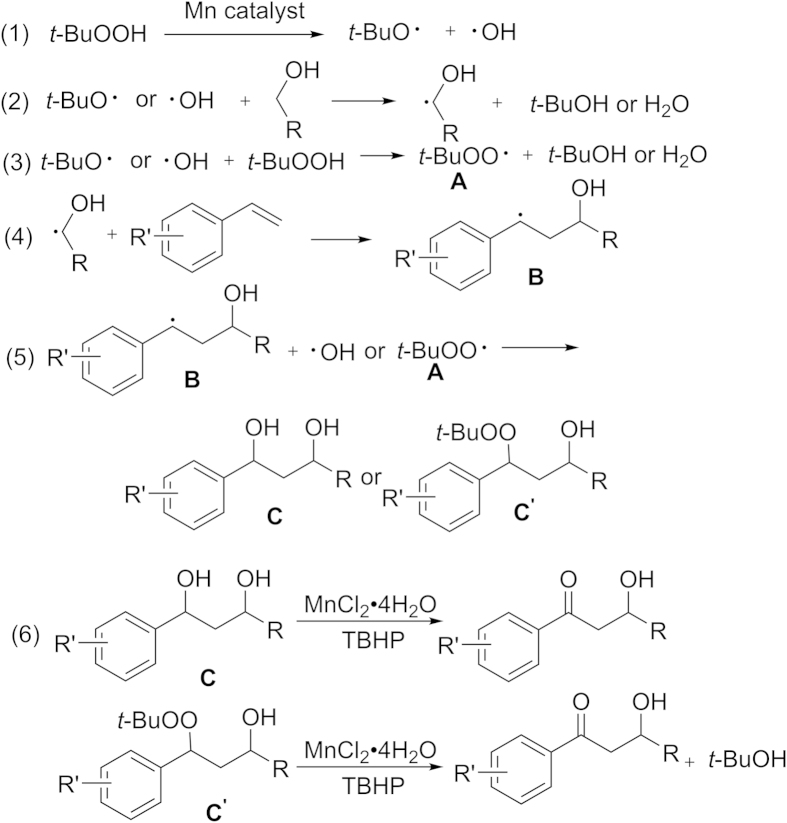
Proposed mechanism.

Because *tert*-butyl hydroperoxide is the most common free radical initiator, and transition metals such as Mn(II) usually promote the generation of this kind of free radicals, we speculated that a free-radical process might be involved in this transformation. When the free-radical scavenger 2,2,6,6-tetramethylpiperidin-1-yloxyl (50 mol%) (TEMPO) was added, the reaction yield dropped and the reaction only gave target product. The preliminary mechanistic studies supported our speculation. According to the related references^21^,^22^,^44^ and our own work, a possible mechanism for this difunctionalization reaction was shown in Fig. 4. Firstly, homolytic cleavage of the free-radical initiator TBHP in the presence of MnCl_2_·4H_2_O produced the alkoxyl radical and hydroxyl radical (eq. 1), and then they abstracted hydrogen from the alcohol or TBHP to form the *α*-hydroxyalkyl radical and free-radical **A** (eqs 2 and 3). Subsequently, intermediate **B** was generated by addition of the *α*-hydroxyalkyl radical to the styrene (eq. 4). Combination of hydroxyl radical or free-radical **A** and intermediate **B** gave the compound **C** or **C′** (eq. 5). Both compound **C** and **C′** were found in the reaction mixtures by high resolution mass spectrum (HRMS) (see supporting information). Finally, the compound **C** or **C′** could be further converted to target molecule under the reaction conditions (eq. 6).

When our paper is being submitted for publication (Angew. Chem. Int. Ed. in Jan. 13, 2015, manuscript ID 201500383), we find that Loh’s paper (as ASAP) was just published in Jan. 2015, which is about copper- and cobalt-catalyzed direct coupling of sp^3^ *α*-carbon of 1,3-enynes and alkenes with alcohol^53^. However, they mainly studied the coupling reaction 1,3-enynes and alcohols.

## Conclusion

In summary, we have developed the first example of Mn-catalyzed difunctionalization reaction of vinylarenes with a series of aliphatic alcohols in the presence of TBHP for the synthesis of the oxyalkylation products. The method exhibits an excellent regioselectivity. This synthetic strategy possesses a single operation to afford useful functionalized molecules directly from cheap and readily available materials. It also enriches the methodology of regioselective oxidative coupling reaction. Considering the simple synthetic operation provided by this methodology, we are convinced that it will find widespread applications in the near future in the synthesis of complex molecules.

## Methods

To a mixture of alkenes (1 mmol), MnCl_2_.4H_2_O (0.1 mmol) and alcohols (10 mL), *tert*-butyl hydroperoxide (3 mmol, 70% in water) was added dropwise at room temperature. The resulting mixture was heated at 70 °C for 12–24 hours. The reaction was monitored by TLC (1:5 ethyl acetate : petroleum ether). When the reaction was finished, the solvent was distilled under reduced pressure. The residue was purified by silica gel column chromatography (1:5 ~ 1:15 ethyl acetate : petroleum ether).

## Additional Information

**How to cite this article**: Zhang, W. *et al.* Manganese-Mediated Coupling Reaction of Vinylarenes and Aliphatic Alcohols. *Sci. Rep.* **5**, 15250; doi: 10.1038/srep15250 (2015).

## Supplementary Material

Supplementary Information

## Figures and Tables

**Figure 1:**
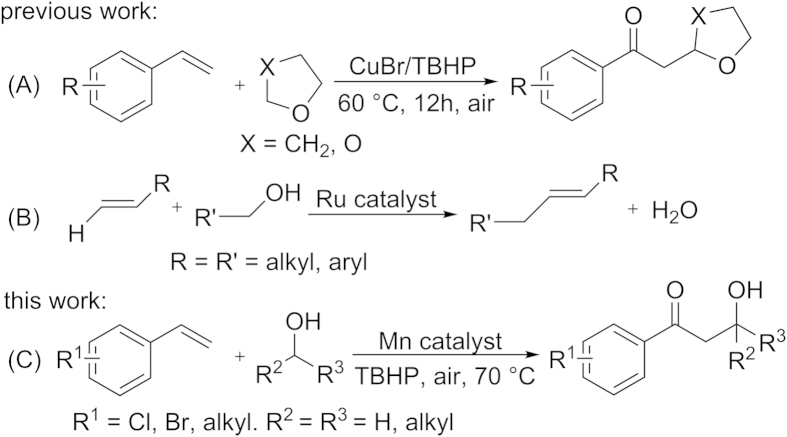
Representative examples and present work.

**Table 1:**
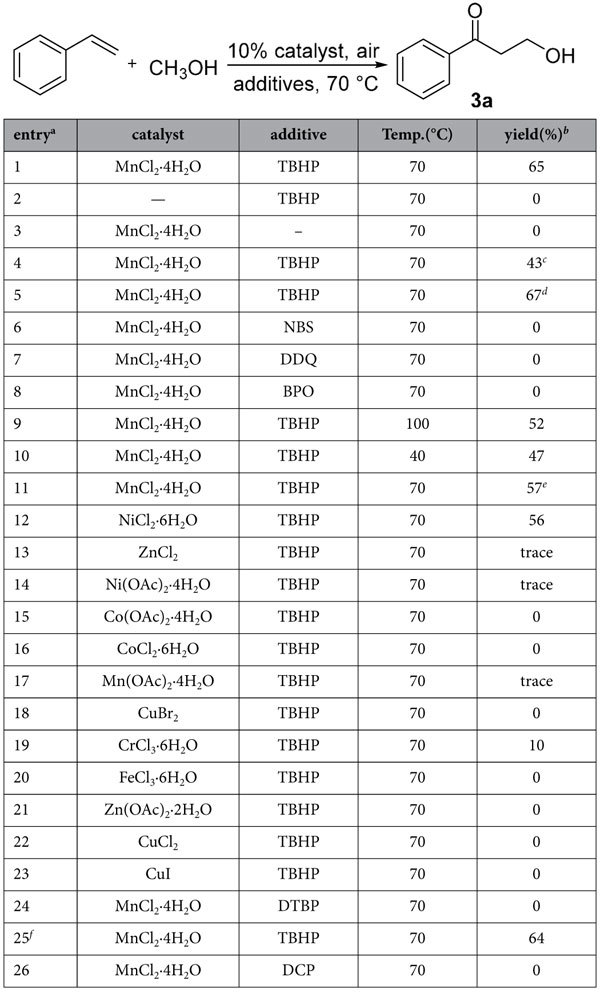
Optimization of reaction condition.

Reaction conditions: styrene (1 mmol), catalyst (0.1 mmol), additive (3 mmol), TBHP = *tert*-butyl hydroperoxide, 70% in water.

^a^Methanol (10 mL).

^b^Isolated yields.

^c^TBHP (2 mmol).

^d^TBHP (5 mmol).

^e^catalyst (0.2 mmol).

^f^Anhydrous TBHP.
