# Supplementary material for: Manganese-Mediated Coupling Reaction of Vinylarenes and Aliphatic Alcohols
Source: Sci Rep. 2015 Oct 16;5:15250. doi: 10.1038/srep15250 (PMC4607950; doi:10.1038/srep15250)
Supplement: Supplementary Information [file srep15250-s1.doc]

Manganese-Mediated Coupling Reaction of Vinylarenes and Aliphatic Alcohols

Wei Zhang,^1^ Nai-Xing Wang,^1^,* Cui-Bing Bai,^1^ Yan-Jing Wang,^1^ Xing-Wang Lan,^1^ Yalan Xing,^2^,* Yi-He Li,^1^ & Jia-Long Wen^1^

^1^*Technical Institute of Physics and Chemistry, Chinese Academy of Sciences, Beijing, 100190, China*
 E-Mail: nxwang@mail.ipc.ac.cn; Tel.: +86-10-82543575; Fax: +86-10-62554670.

^2^*Department of Chemistry, William Paterson University of New Jersey, 300 Pompton Road, Wayne, New Jersey 07470, United States.*

E-Mail: xingy@wpunj.edu

**Table of Contents:**

1. General S2

2. General Procedure S2

3. Characterization data for **3a – 3u** S3- S7

4. ^1^H and ^13^C NMR spectra for **3a – 3u**  S8- S28

5. HRMS spectra for **3a – 3u,** intermediate **C** and **C’**  S29- S40

1. **General**

Reaction process was tracked by TLC analysis at 254 nm. NMR spectroscopy was run on 400 MHz spectrometer operating at 400 MHz (^1^H NMR) and 100 MHz (^13^C NMR). TMS was used as an internal standard and CDCl_3_ was used as the solvent for all samples. ^1^H NMR data were reported as follows: chemical shifts in ppm downfield from tetramethylsilane, multiplicity (s = singlet, d = doublet, t = triplet, q = quartet and m = multiplet), coupling constant = *J*. High-resolution mass spectral analyses (HRMS) were measured using ESI ionization. All reagents are used directly and without further purification. Mass spectra were in general recorded on a Bruker Microflex mass spectrometer.

1. **General Procedure:**

To a mixture of alkenes (1 mmol), MnCl_2_.4H_2_O (0.1 mmol) and alcohols (10 mL), *tert*-butyl hydroperoxide (3 mmol, 70% in water) was added dropwise at room temperature. The resulting mixture was heated at 70 ºC for 12-24 hours. The reaction was monitored by TLC (1:5 ethyl acetate : petroleum ether). When the reaction was finished, the solvent was distilled under reduced pressure. The residue was purified by silica gel column chromatography (1:5 ~ 1:15 ethyl acetate : petroleum ether).

1. **Characterization data for** **3a** **–** **3u**

**3-hydroxy-1-phenylpropan-1-one**

Yellow oil; R*_f_* = 0.10 (1:5 ethyl acetate : petroleum ether); 97.6 mg; 65% yield; ^1^H NMR (400 MHz, CDCl_3_) δ 7.97 (d, *J* = 8.0 Hz, 2H), 7.59 (t, *J* = 6.0 Hz, 1H), 7.48 (t, *J* = 8.0 Hz, 2H), 4.04 (t, *J* = 4.0 Hz, 2H), 3.24 (t, *J* = 4.0 Hz, 2H), 2.72 (s, 1H). ^13^C NMR (100 MHz, CDCl_3_) δ 200.46, 136.82, 133.56, 128.77, 128.17, 58.12, 40.63. HRMS (ESI^+^) calcd for C_9_H_10_O_2_, [M+H]^+^: 151.0754. Found 151.0753.

**3-hydroxy-1-phenylbutan-1-one**

Yellow oil; R*_f_* = 0.20 (1:5 ethyl acetate : petroleum ether); 110 mg; 67% yield; ^1^H NMR (400 MHz, CDCl_3_) δ 7.95 (d, *J* = 8.0 Hz, 2H), 7.57 (t, *J* = 6.0 Hz, 1H), 7.46 (t, *J* = 8.0 Hz, 2H), 4.40 (s, 1H), 3.39 (s, 1H), 3.15 (dd, *J* = 16.0, 4.0 Hz, 1H), 3.05 (dd, *J* = 18.0, 10.0 Hz, 1H), 1.30 (d, *J* = 4.0 Hz, 3H). ^13^C NMR (100 MHz, CDCl_3_) δ 200.75, 136.77, 133.50, 128.66, 128.06, 64.03, 46.56, 22.48. HRMS (ESI^+^) calcd for C_10_H_12_O_2_, [M+Na]^+^: 187.0730. Found 187.0730.

**3-hydroxy-3-methyl-1-phenylbutan-1-one**

Yellow oil; R*_f_* = 0.29 (1:5 ethyl acetate : petroleum ether); 126.5 mg; 71% yield; ^1^H NMR (400 MHz, CDCl_3_) δ 7.95 (d, *J* = 8.0 Hz, 2H), 7.58 (t, *J* = 8.0 Hz, 1H), 7.47 (t, *J* = 8.0 Hz, 2H), 4.15 (s, 1H), 3.14 (s, 2H), 1.35 (s, 6H). ^13^C NMR (100 MHz, CDCl_3_) δ 201.71, 137.33, 133.56, 128.68, 128.08, 69.89, 48.64, 29.57. HRMS (ESI^+^) calcd for C_11_H_14_O_2_, [M+H]^+^: 179.1067. Found 179.1064.

**3-hydroxy-1-(m-tolyl)propan-1-one**

Yellow oil; R*_f_* = 0.12 (1:5 ethyl acetate : petroleum ether); 100.2 mg; 61% yield; ^1^H NMR (400 MHz, CDCl_3_) δ 7.76 (d, *J* = 8.0 Hz, 1H), 7.41 – 7.33 (m, 2H), 4.03 (t, *J* = 6.0 Hz, 1H), 3.22 (t, *J* = 4.0 Hz, 2H), 2.41 (s, 3H). ^13^C NMR (100 MHz, CDCl_3_) δ 200.73, 138.60, 136.96, 134.32, 128.67(2C), 125.43, 58.25, 40.63, 21.40. HRMS (ESI^+^) calcd for C_10_H_12_O_2_, [M+Na]^+^: 187.0730. Found 187.0731.

**3-hydroxy-1-(m-tolyl)butan-1-one**

Yellow oil; R*_f_* = 0.18 (1:5 ethyl acetate : petroleum ether); 112.3 mg; 63% yield; ^1^H NMR (400 MHz, CDCl_3_) δ 7.74 (d, *J* = 8.0 Hz, 2H), 7.39 – 7.32 (m, 2H), 4.39 (t, *J* = 6.0 Hz, 1H), 3.49 (s, 1H), 3.14 (dd, *J* = 16.0, 4.0 Hz, 1H), 3.03 (dd, *J* = 16.0, 8.0 Hz, 1H), 2.40 (s, 3H), 1.29 (d, *J* = 8.0 Hz, 3H). ^13^C NMR (100 MHz, CDCl_3_) δ 200.97, 138.46, 136.79, 134.26, 128.55, 128.53, 125.31, 64.06, 46.59, 22.47, 21.32. HRMS (ESI^+^) calcd for C_11_H_14_O_2_, [M+Na]^+^: 201.0881. Found 201.0881.

**3-hydroxy-3-methyl-1-(m-tolyl)butan-1-one**

Yellow oil; R*_f_* = 0.28 (1:5 ethyl acetate : petroleum ether); 126.9 mg; 66% yield; ^1^H NMR (400 MHz, CDCl_3_) δ 7.75 (t, *J* = 6.0 Hz, 2H), 7.41 – 7.34 (m, 2H), 4.22 (s, 1H), 3.14 (s, 2H), 2.42 (s, 3H), 1.34 (s, 6H). ^13^C NMR (100 MHz, CDCl_3_) δ 201.35, 137.97, 137.01, 133.71, 128.02, 128.00, 124.80, 69.34, 48.21, 29.04, 20.74. HRMS (ESI^+^) calcd for C_12_H_16_O_2_, [M+Na]^+^: 215.1043. Found 215.1038.

**1-(3-bromophenyl)-3-hydroxypropan-1-one**

Yellow oil; R*_f_* = 0.06 (1:5 ethyl acetate : petroleum ether); 116.8 mg; 51% yield; ^1^H NMR (400 MHz, CDCl_3_) δ 8.02 (t, *J* = 1.8 Hz, 1H), 7.81 (d, *J* = 8.0 Hz, 1H), 7.66 – 7.63 (m, 1H), 7.29 (t, *J* = 8.0 Hz, 1H), 3.96 (t, *J* = 5.2 Hz, 2H), 3.13 (t, *J* = 5.4 Hz, 2H), 2.51 (s, 1H). ^13^C NMR (100 MHz, CDCl_3_) δ 198.33, 137.99, 135.75, 130.63, 129.72, 126.03, 122.56, 57.38, 40.11. HRMS (ESI^+^) calcd for C_9_H_9_BrO_2_, [M+Na]^+^: 250.9678. Found 250.9674.

**1-(3-bromophenyl)-3-hydroxybutan-1-one**

Yellow oil; R*_f_* = 0.15 (1:5 ethyl acetate : petroleum ether); 133.7 mg; 55% yield; ^1^H NMR (400 MHz, CDCl_3_) δ 8.08 (t, *J* = 1.8 Hz, 1H), 7.89 – 7.86 (m, 1H), 7.72 – 7.70 (m, 1H), 7.36 (t, *J* = 8.0 Hz, 1H), 4.43 – 4.39 (m, 1H), 3.20 (s, 1H), 3.13 (dd, *J* = 17.8, 3.0 Hz, 1H), 3.04 (dd, *J* = 17.8, 8.6 Hz, 1H), 1.31 (d, *J* = 6.4 Hz, 3H). ^13^C NMR (100 MHz, CDCl_3_) δ 198.62, 138.07, 135.74, 130.63, 129.70, 126.04, 122.54, 63.44, 46.23, 21.98. HRMS (ESI^+^) calcd for C_10_H_11_BrO_2_, [M+Na]^+^: 264.9835. Found 264.9831.

**3-hydroxy-1-(p-tolyl)propan-1-one**

Yellow oil; R*_f_* = 0.12 (1:5 ethyl acetate : petroleum ether); 114.9 mg; 70% yield; ^1^H NMR (400 MHz, CDCl_3_) δ 7.84 (d, *J* = 8.0 Hz, 2H), 7.24 (d, *J* = 8.0 Hz, 2H), 4.01 (t, *J* = 6.0 Hz, 2H), 3.25 (s, 1H), 3.18 (t, *J* = 6.0 Hz, 2H), 2.39 (s, 3H). ^13^C NMR (100 MHz, CDCl_3_) δ 199.96, 144.27, 134.27, 129.30, 128.18, 58.07, 40.39, 21.61. HRMS (ESI^+^) calcd for C_10_H_12_O_2_, [M+H]^+^: 165.0910. Found 165.0910.

**3-hydroxy-1-(p-tolyl)butan-1-one**

Yellow oil; R*_f_* = 0.22 (1:5 ethyl acetate : petroleum ether); 130.1 mg; 73% yield; ^1^H NMR (400 MHz, CDCl_3_) δ 7.84 (d, *J* = 8.0 Hz, 2H), 7.25 (d, *J* = 8.0 Hz, 2H), 4.41 – 4.36 (m, 1H), 3.51 (s, 1H), 3.13 (dd, *J* = 16.0, 4.0 Hz, 1H), 3.01 (dd, *J* = 16.0, 8.0 Hz, 1H), 2.40 (s, 3H), 1.29 (d, *J* = 4.0 Hz, 3H). ^13^C NMR (100 MHz, CDCl_3_) δ 200.42, 144.39, 134.31, 129.34, 128.20, 64.10, 46.39, 22.47, 21.63. HRMS (ESI^+^) calcd for C_11_H_14_O_2_, [M+H]^+^: 179.1067. Found 179.1063.

**3-hydroxy-3-methyl-1-(p-tolyl)butan-1-one**

Yellow oil; R*_f_* = 0.33 (1:5 ethyl acetate : petroleum ether); 153.8 mg; 80% yield; ^1^H NMR (400 MHz, CDCl_3_) δ 7.85 (d, *J* = 8.0 Hz, 2H), 7.25 (d, *J* = 8.0 Hz, 2H), 3.12 (s, 2H), 2.40 (s, 3H), 1.34 (s, 6H). ^13^C NMR (100 MHz, CDCl_3_) δ 201.40, 144.51, 134.85, 129.36, 128.25, 70.02, 48.38, 29.53, 21.62. HRMS (ESI^+^) calcd for C_12_H_16_O_2_, [M+H]^+^: 193.1223. Found 193.1223.

**1-(4-chlorophenyl)-3-hydroxypropan-1-one**

Yellow oil; R*_f_* = 0.06 (1:5 ethyl acetate : petroleum ether); 112.6 mg; 61% yield; ^1^H NMR (400 MHz, CDCl_3_) δ 7.91 (d, *J* = 8.0 Hz, 2H), 7.45 (d, *J* = 8.0 Hz, 2H), 4.04 (t, *J* = 6.0 Hz, 2H), 3.20 (t, *J* = 6.0 Hz, 2H), 2.81 (s, 1H). ^13^C NMR (100 MHz, CDCl_3_) δ 198.48, 139.48, 134.61, 128.91, 128.48, 57.44, 39.93. HRMS (ESI^+^) calcd for C_9_H_9_ClO_2_, [M+Na]^+^: 207.0183. Found 207.0181.

**1-(4-chlorophenyl)-3-hydroxybutan-1-one**

Yellow oil; R*_f_* = 0.15 (1:5 ethyl acetate : petroleum ether); 135.1 mg; 68% yield; ^1^H NMR (400 MHz, CDCl_3_) δ 7.89 (d, *J* = 8.0 Hz, 2H), 7.44 (d, *J* = 12.0 Hz, 2H), 4.43 – 4.39 (m, 1H), 3.54 (s, 1H), 3.11 (dd, *J* = 16.0, 4.0 Hz, 1H), 3.04 (dd, *J* = 16.0, 8.0 Hz, 1H), 1.30 (d, *J* = 8.0 Hz, 3H). ^13^C NMR (100 MHz, CDCl_3_) δ 199.42, 140.01, 135.04, 129.50, 129.00, 63.99, 46.56, 22.48. HRMS (ESI^+^) calcd for C_10_H_11_ClO_2_, [M+Na]^+^: 221.0340. Found 221.0330.

**1-(4-chlorophenyl)-3-hydroxy-3-methylbutan-1-one**

Yellow oil; R*_f_* = 0.21 (1:5 ethyl acetate : petroleum ether); 155.2 mg; 73% yield; ^1^H NMR (400 MHz, CDCl_3_) δ 7.89 (d, *J* = 8.0 Hz, 2H), 7.45 (d, *J* = 8.0 Hz, 2H), 3.99 (s, 1H), 3.12 (s, 2H), 1.35 (s, 6H). ^13^C NMR (100 MHz, CDCl_3_) δ 199.65, 139.54, 135.22, 128.96, 128.46, 69.32, 48.28, 29.05. HRMS (ESI^+^) calcd for C_11_H_13_ClO_2_, [M+Na]^+^: 235.0496. Found 235.0487.

**1-(4-(tert-butyl)phenyl)-3-hydroxypropan-1-one**

Yellow oil; R*_f_* = 0.13 (1:5 ethyl acetate : petroleum ether); 123.7 mg; 60% yield; ^1^H NMR (400 MHz, CDCl_3_) δ 7.90 (d, *J* = 8.0 Hz, 2H), 7.47 (d, *J* = 8.0 Hz, 2H), 4.02 (t, *J* = 6.0 Hz, 2H), 3.29 (s, 1H), 3.21 (t, *J* = 6.0 Hz, 2H), 1.33 (s, 9H). ^13^C NMR (100 MHz, CDCl_3_) δ 200.12, 157.30, 134.18, 128.08, 125.62, 77.42, 77.10, 76.79, 58.15, 40.36, 35.14, 31.06. HRMS (ESI^+^) calcd for C_13_H_18_O_2_, [M+H]^+^: 207.1380. Found 207.1383.

**1-(4-(tert-butyl)phenyl)-3-hydroxybutan-1-one**

Yellow oil; R*_f_* = 0.27 (1:5 ethyl acetate : petroleum ether); 136.6 mg; 62% yield; ^1^H NMR (400 MHz, CDCl_3_) δ 7.90 (d, *J* = 8.8 Hz, 2H), 7.48 (d, *J* = 8.4 Hz, 2H), 4.44 – 4.36 (m, 1H), 3.54 (s, 1H), 3.15 (dd, *J* = 17.4, 3.0 Hz, 1H), 3.03 (dd, *J* = 17.6, 8.8 Hz, 1H), 1.34 (s, 9H), 1.30 (d, *J* = 6.4 Hz, 3H). ^13^C NMR (100 MHz, CDCl_3_) δ 200.29, 157.27, 134.37, 128.06, 125.55, 64.18, 46.46, 35.08, 31.01, 22.52. HRMS (ESI^+^) calcd for C_14_H_20_O_2_, [M+H]^+^: 221.1536. Found 221.1533.

**1-(4-bromophenyl)-3-hydroxypropan-1-one**

Yellow oil; R*_f_* = 0.12 (1:5 ethyl acetate : petroleum ether); 128.3 mg; 56% yield; ^1^H NMR (400 MHz, CDCl_3_) δ 7.81 (d, *J* = 8.0 Hz, 2H), 7.60 (d, *J* = 8.0 Hz, 2H), 4.02 (t, *J* = 4.0 Hz, 2H), 3.18 (t, *J* = 4.0 Hz, 2H), 2.85 (s, 1H). ^13^C NMR (100 MHz, CDCl_3_) δ 199.24, 135.42, 132.00, 129.58, 128.70, 57.87, 40.50. HRMS (ESI^+^) calcd for C_9_H_9_BrO_2_, [M+H]^+^: 228.9859. Found 228.9852.

**1-(4-bromophenyl)-3-hydroxybutan-1-one**

Yellow oil; R*_f_* = 0.16 (1:5 ethyl acetate : petroleum ether); 167.7 mg; 69% yield; ^1^H NMR (400 MHz, CDCl_3_) δ 7.81 (d, *J* = 8.4 Hz, 2H), 7.60 (d, *J* = 8.4 Hz, 2H), 4.44 – 4.36 (m, 1H), 3.30 (s, 1H), 3.11 (dd, *J* = 17.8, 3.0 Hz, 1H), 3.02 (dd, *J* = 17.6, 8.8 Hz, 1H), 1.29 (d, *J* = 6.4 Hz, 3H). ^13^C NMR (100 MHz, CDCl_3_) δ 199.12, 134.90, 131.48, 129.05, 128.25, 63.42, 45.99, 21.92. HRMS (ESI^+^) calcd for C_10_H_11_BrO_2_, [M+H]^+^: 243.0015. Found 243.0012.

**1-(4-bromophenyl)-3-hydroxy-3-methylbutan-1-one**

Yellow oil; R*_f_* = 0.22 (1:5 ethyl acetate : petroleum ether); 192.8 mg; 75% yield; ^1^H NMR (400 MHz, CDCl_3_) δ 7.78 (dd, *J* = 8.6, 1.8 Hz, 2H), 7.58 (dd, *J* = 8.6, 2.2 Hz, 2H), 3.97 (s, 1H), 3.09 (s, 2H), 1.32 (s, 6H). ^13^C NMR (100 MHz, CDCl_3_) δ 199.93, 135.45, 131.46, 129.08, 128.29, 69.37, 48.17, 29.02. HRMS (ESI^+^) calcd for C_11_H_13_BrO_2_, [M+H]^+^: 257.0172. Found 257.0182.

**2-(1-hydroxycyclohexyl)-1-phenylethan-1-one**

Yellow oil; R*_f_* = 0.47 (1:5 ethyl acetate : petroleum ether); 126.6 mg; 58% yield; ^1^H NMR (400 MHz, CDCl_3_) δ 7.95 (t, *J* = 4.0 Hz, 2H), 7.59 (t, *J* = 8.0 Hz, 1H), 7.48 (t, *J* = 8.0 Hz, 2H), 3.99 (s, 1H), 3.12 (s, 2H), 1.79 – 1.66 (m, 5H), 1.49 – 1.42 (m, 5H). ^13^C NMR (100 MHz, CDCl_3_) δ 201.29, 137.19, 132.89, 128.10, 127.56, 70.44, 47.28, 37.35, 25.26, 21.47. HRMS (ESI^+^) calcd for C_14_H_18_O_2_, [M+Na]^+^: 241.1199. Found 241.1200.

**1-(3-bromophenyl)-3-hydroxy-3-methylbutan-1-one**

Yellow oil; R*_f_* = 0.25 (1:5 ethyl acetate : petroleum ether); 146.5 mg; 57% yield; ^1^H NMR (400 MHz, CDCl_3_) δ 8.07 (s, 1H), 7.87 (d, *J* = 8.0 Hz, 1H), 7.71 (d, *J* = 4.0 Hz, 1H), 7.36 (t, *J* = 8.0 Hz, 1H), 3.12 (s, 2H), 1.35 (s, 6H). ^13^C NMR (100 MHz, CDCl_3_) δ 200.17, 138.99, 136.40, 131.17, 130.29, 126.62, 123.10, 69.88, 48.87, 29.55. HRMS (ESI^+^) calcd for C_11_H_13_BrO_2_, [M-H]^-^: 255.0026. Found 255.0019.

1. **^1^H and ^13^C NMR spectra for 3a – 3u**


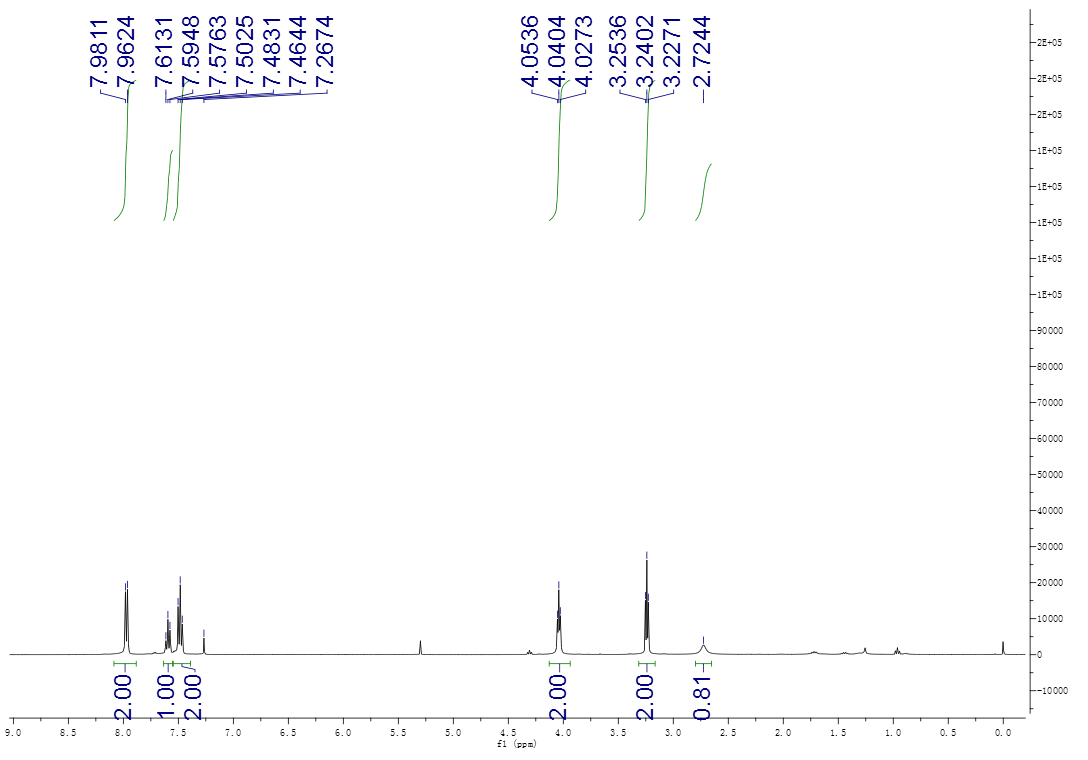


Fig. 1 ^1^H NMR spectrum of product **3a**


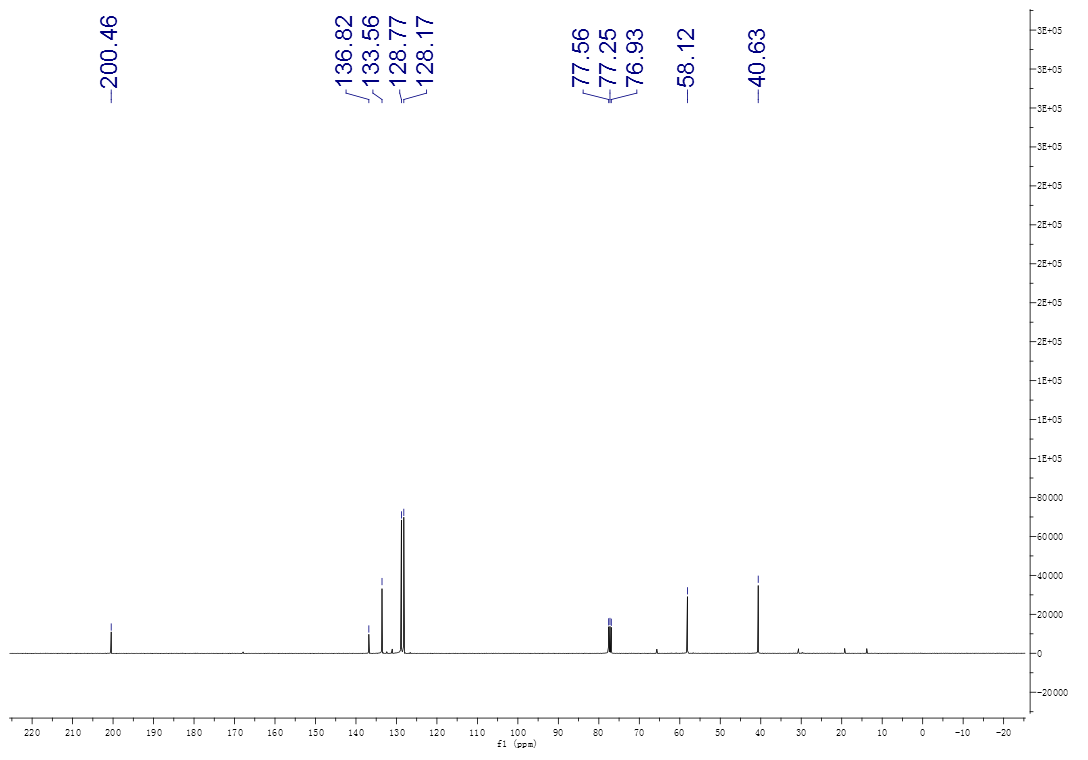


Fig. 2 ^13^C NMR spectrum of product **3a**


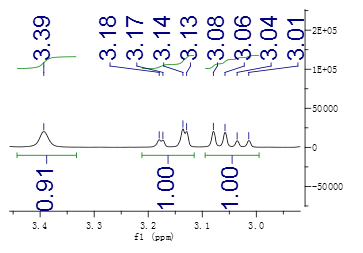

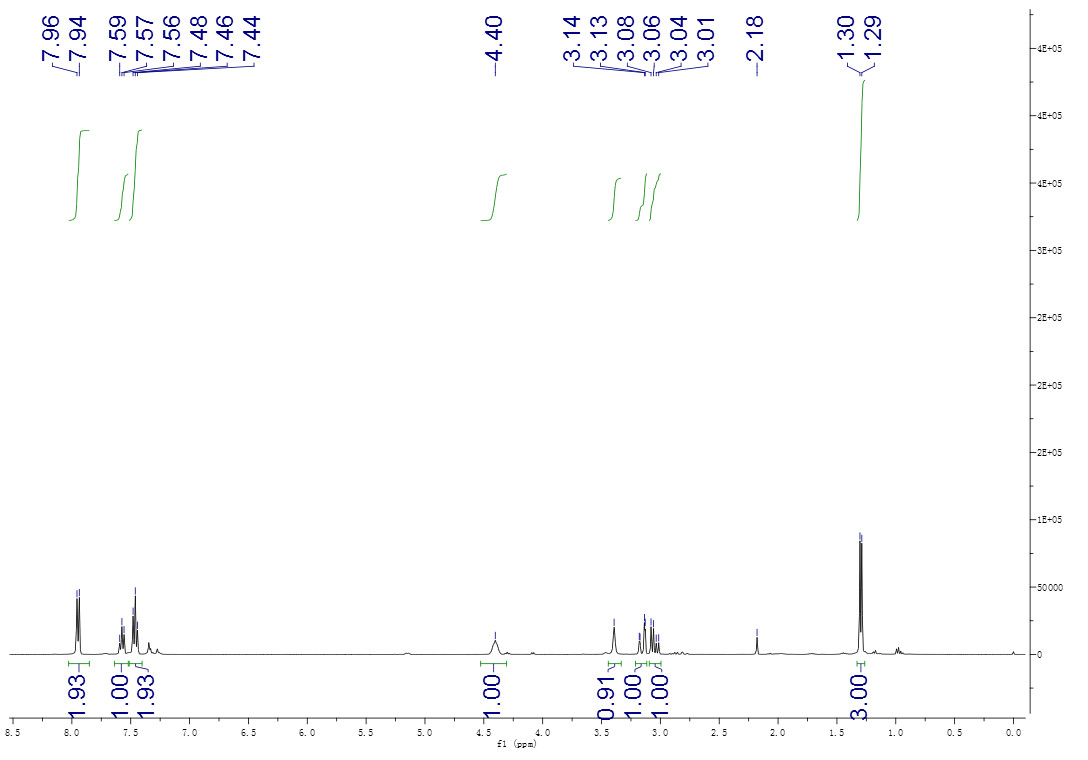


Fig. 3 ^1^H NMR spectrum of product **3b**


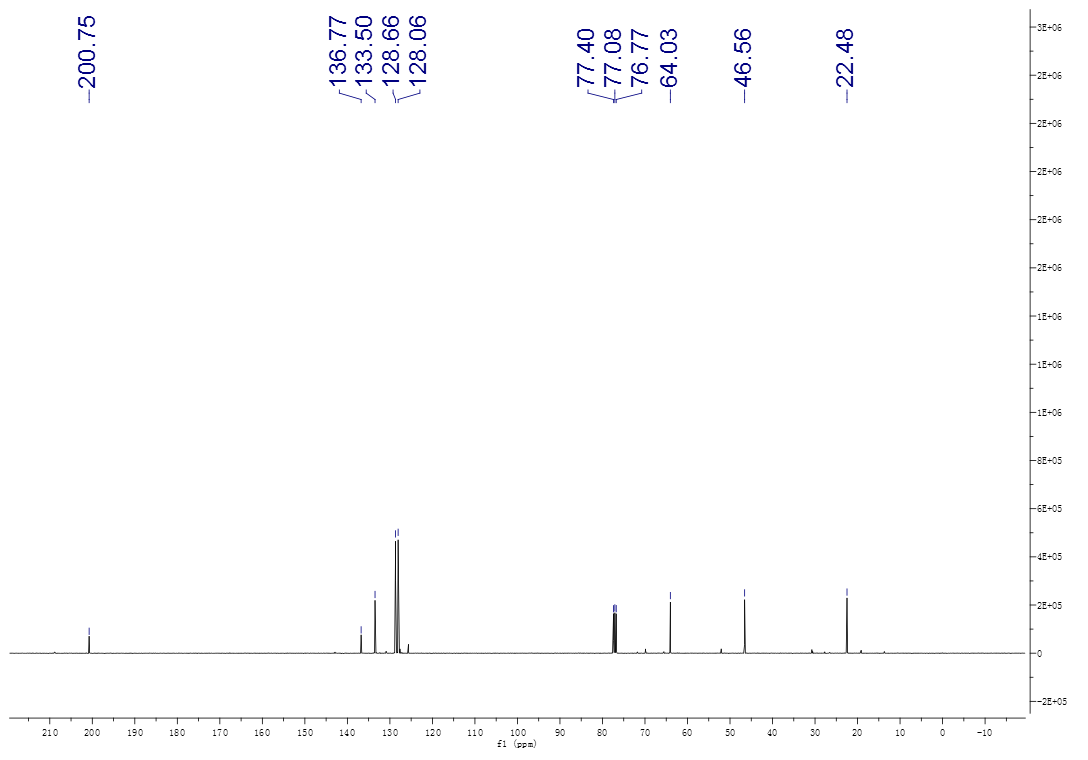


Fig. 4 ^13^C NMR spectrum of product **3b**


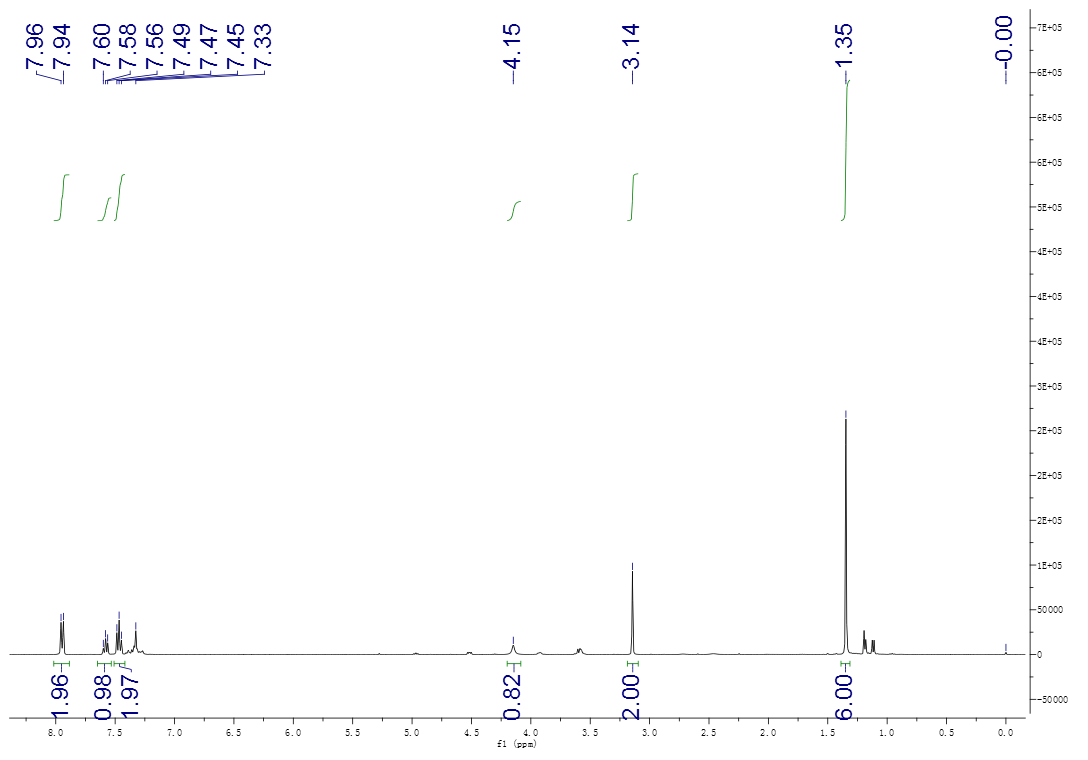


Fig. 5 ^1^H NMR spectrum of product **3c**


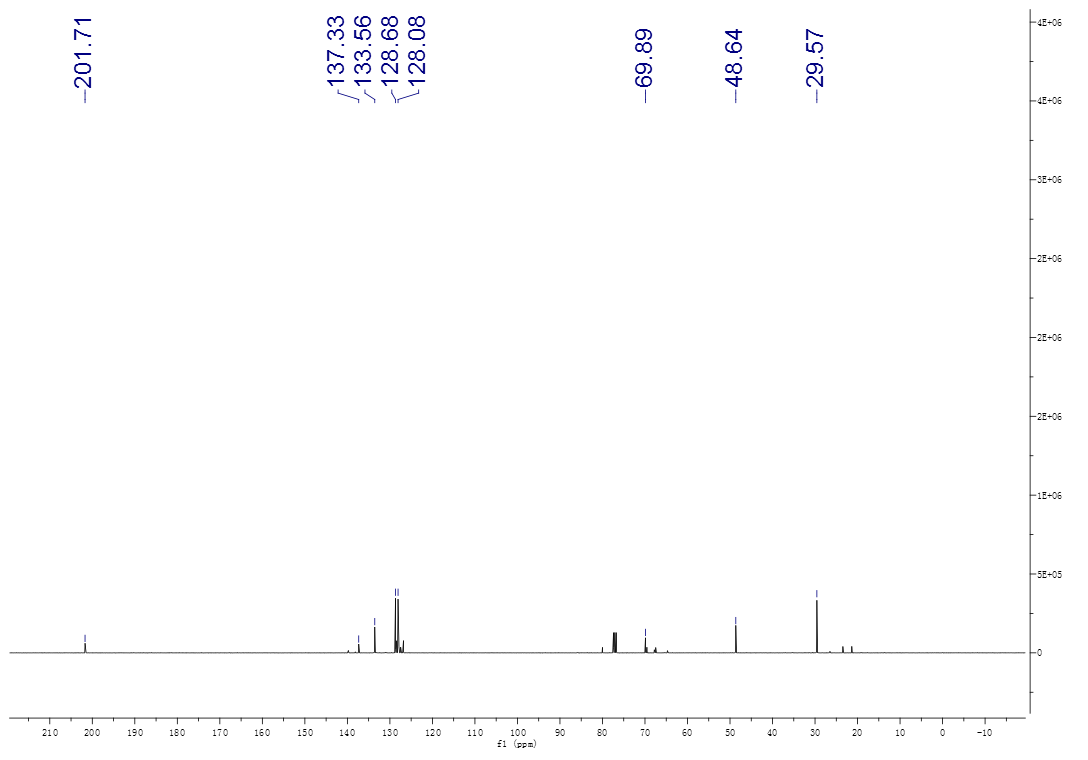


Fig. 6 ^13^C NMR spectrum of product **3c**


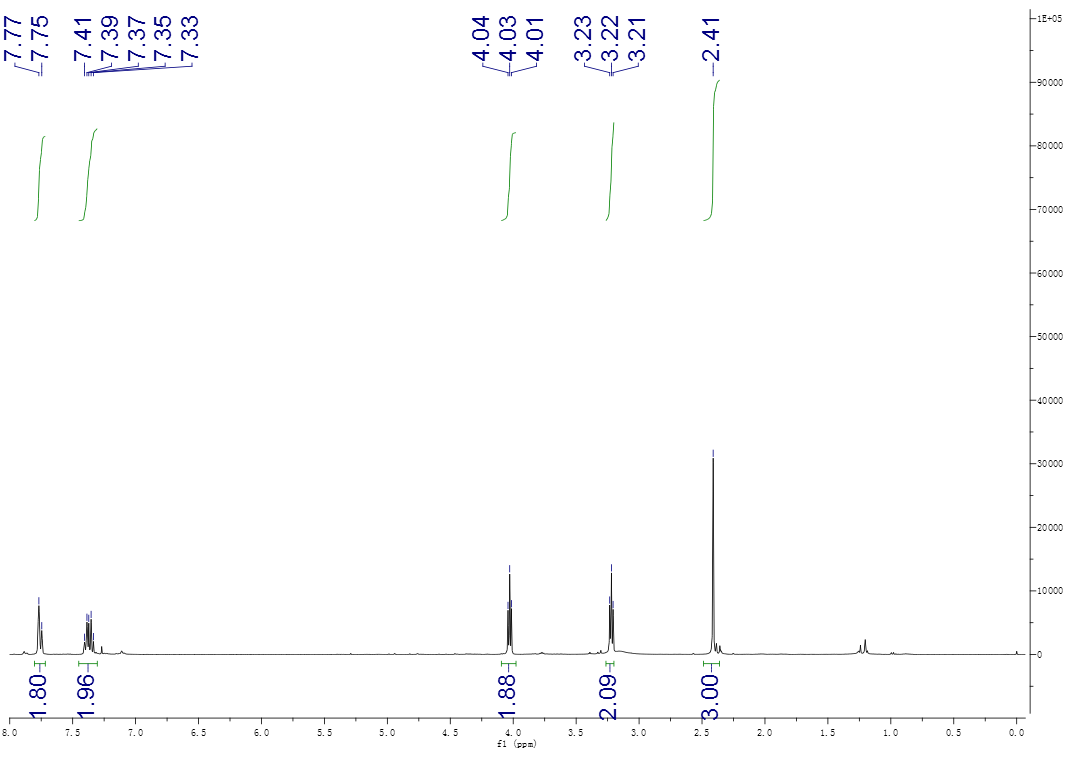


Fig. 7 ^1^H NMR spectrum of product **3d**


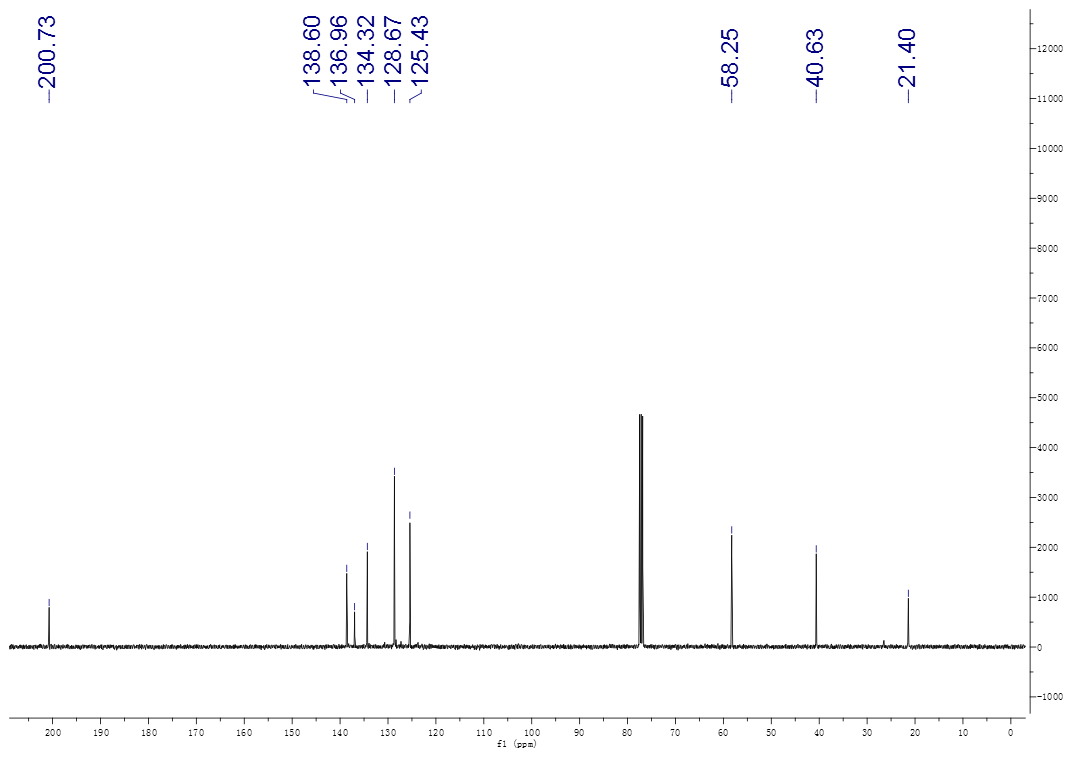


Fig. 8 ^13^C NMR spectrum of product **3d**


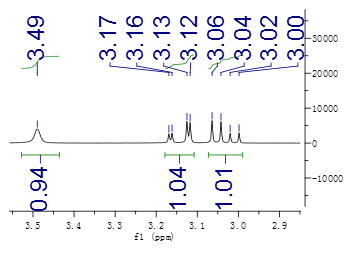

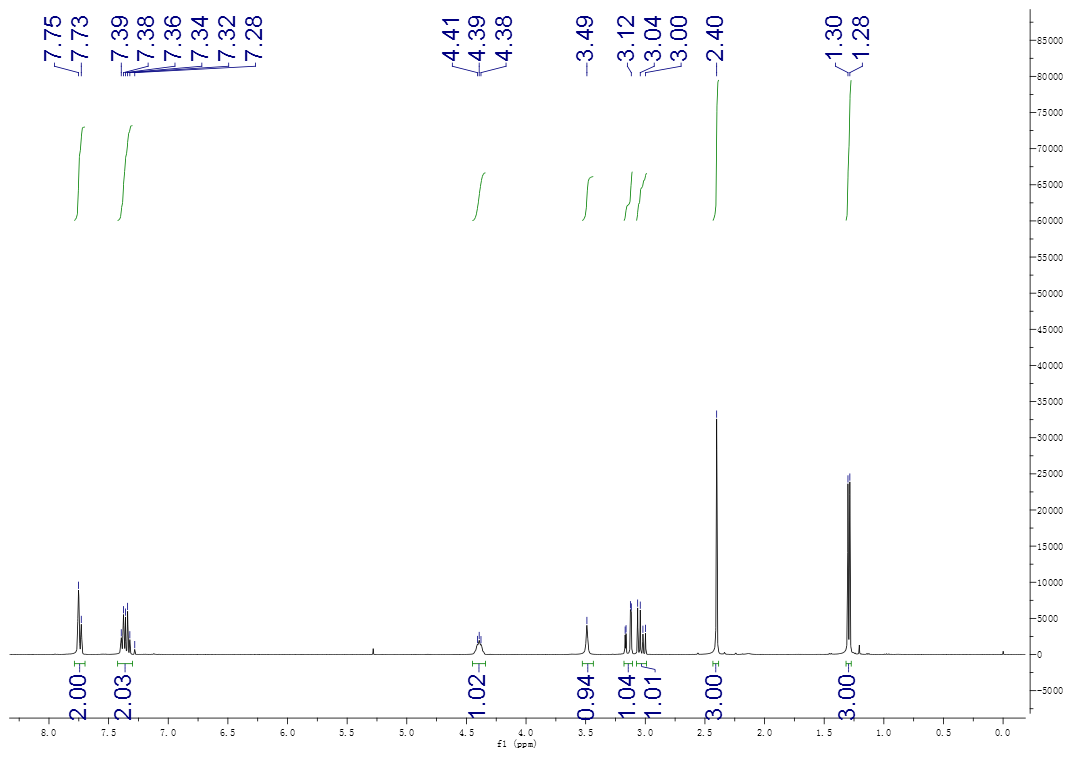


Fig. 9 ^1^H NMR spectrum of product **3e**


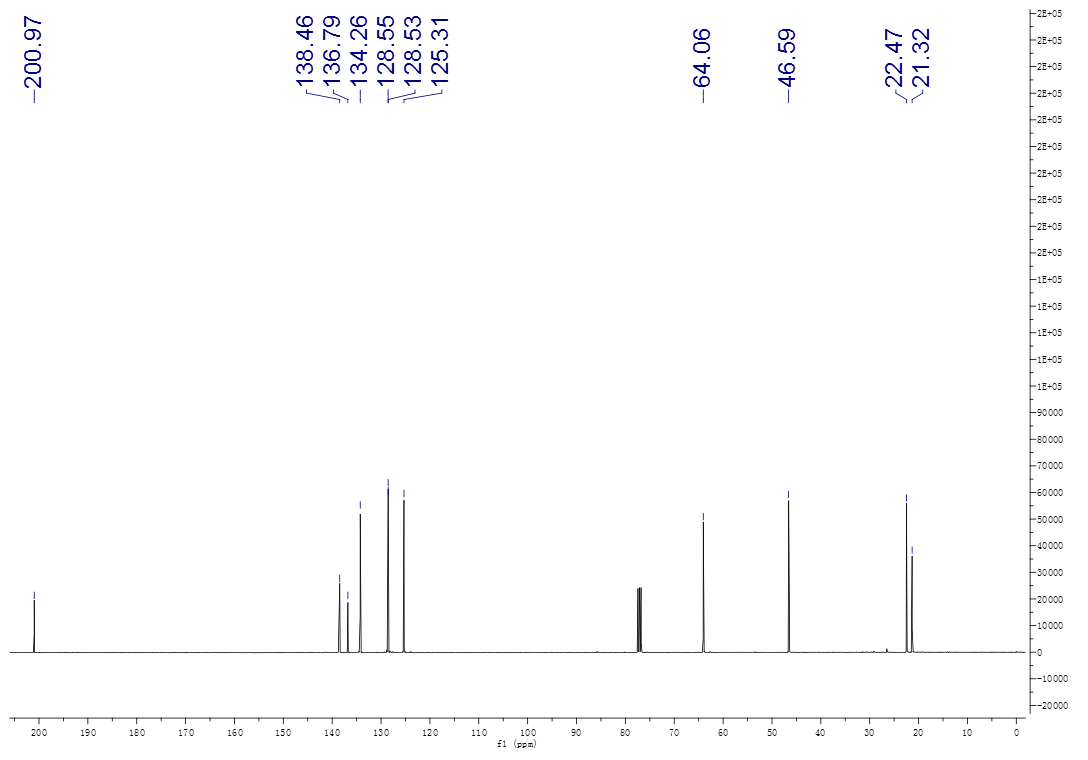


Fig. 10 ^13^C NMR spectrum of product **3e**


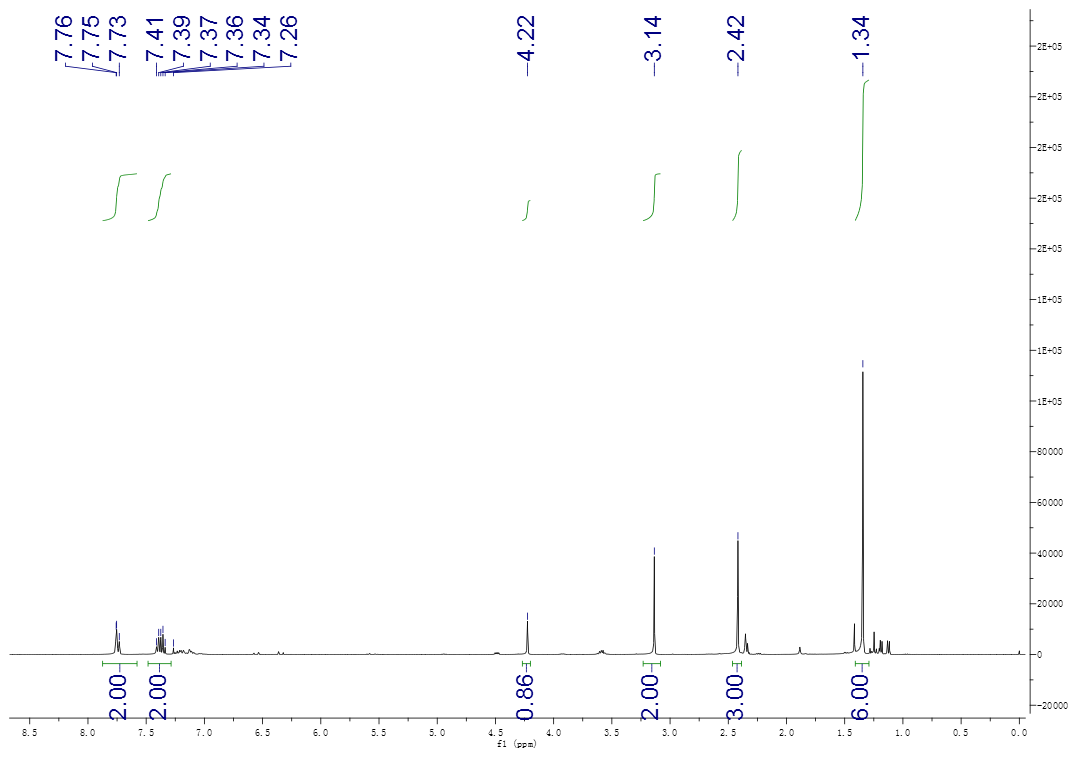


Fig. 11 ^1^H NMR spectrum of product **3f**


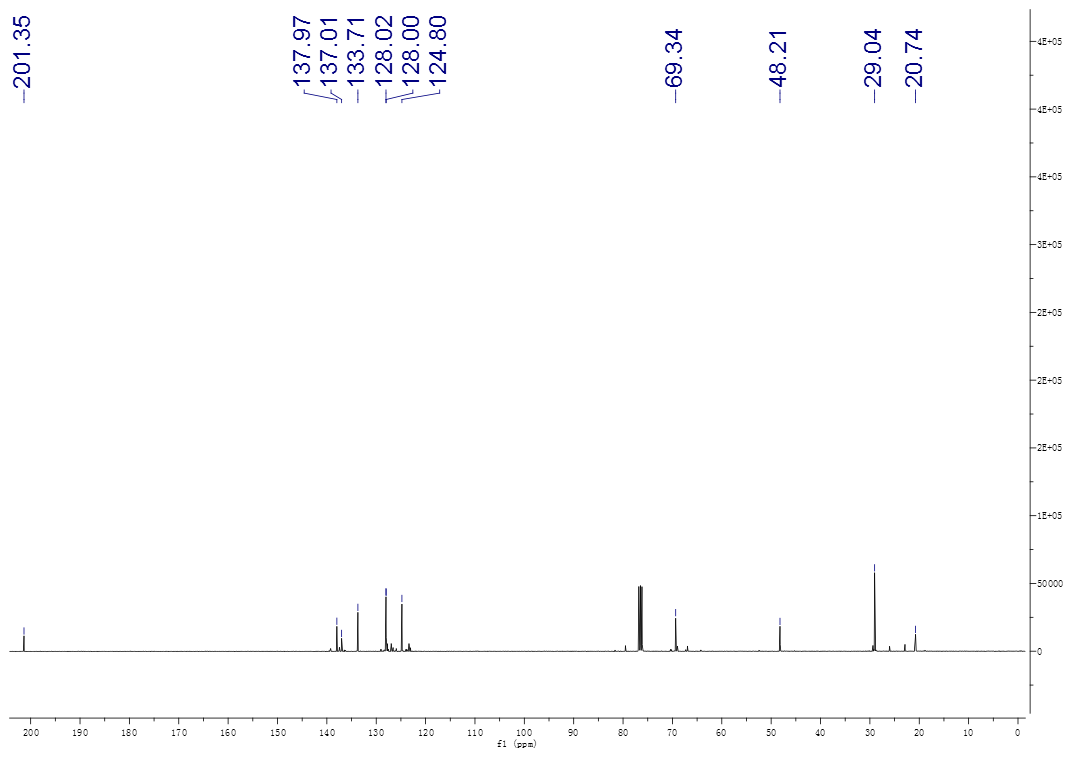


Fig. 12 ^13^C NMR spectrum of product **3f**


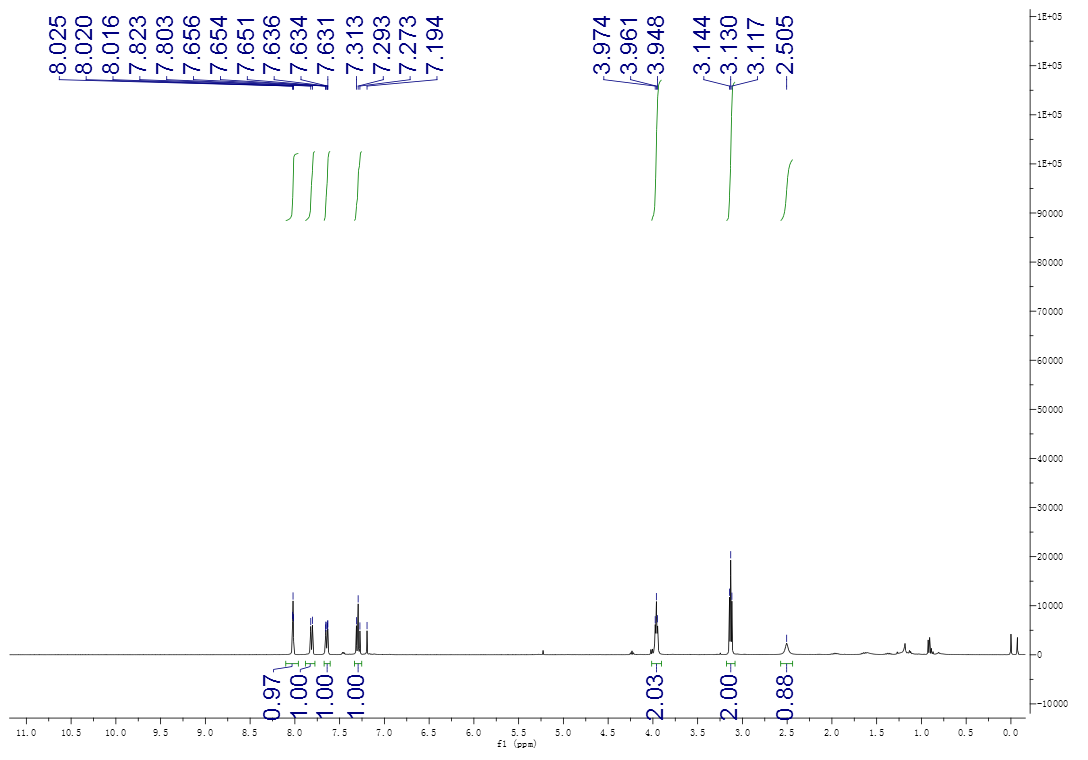


Fig. 13 ^1^H NMR spectrum of product **3g**


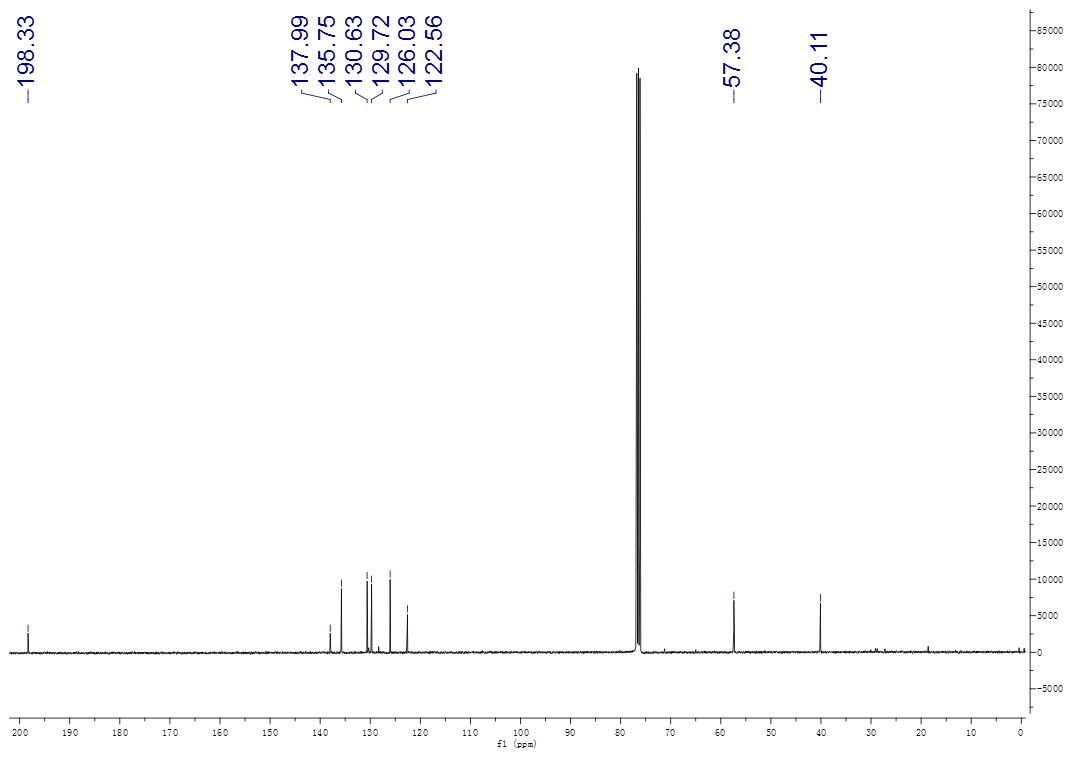


Fig. 14 ^13^C NMR spectrum of product **3g**


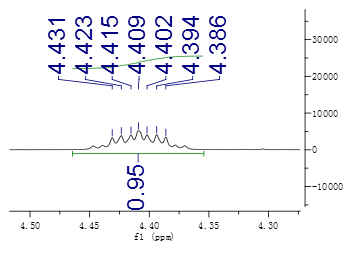

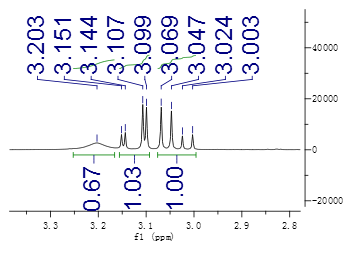

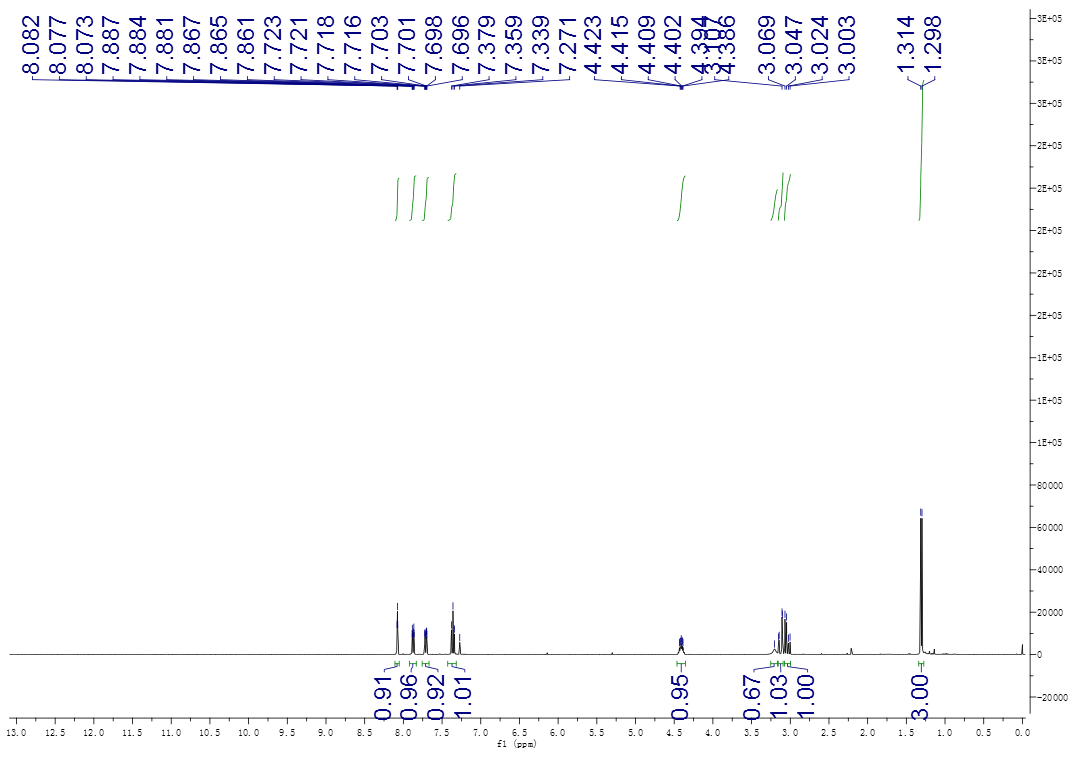


Fig. 15 ^1^H NMR spectrum of product **3h**


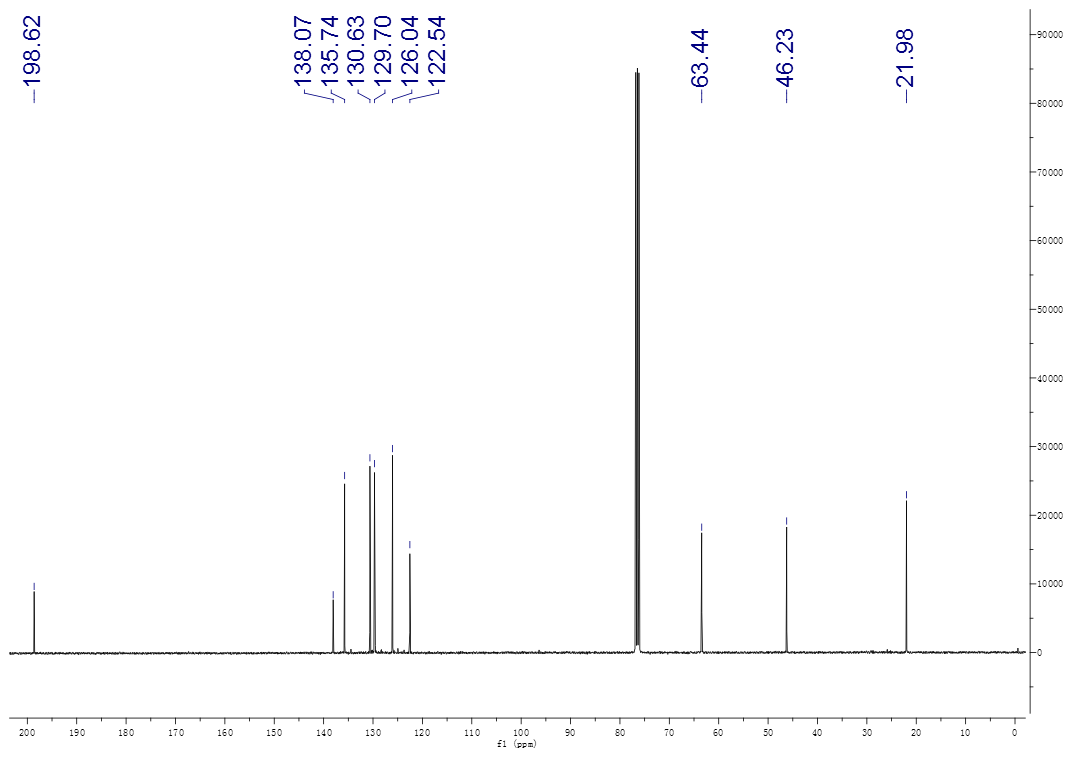


Fig. 16 ^13^C NMR spectrum of product **3h**


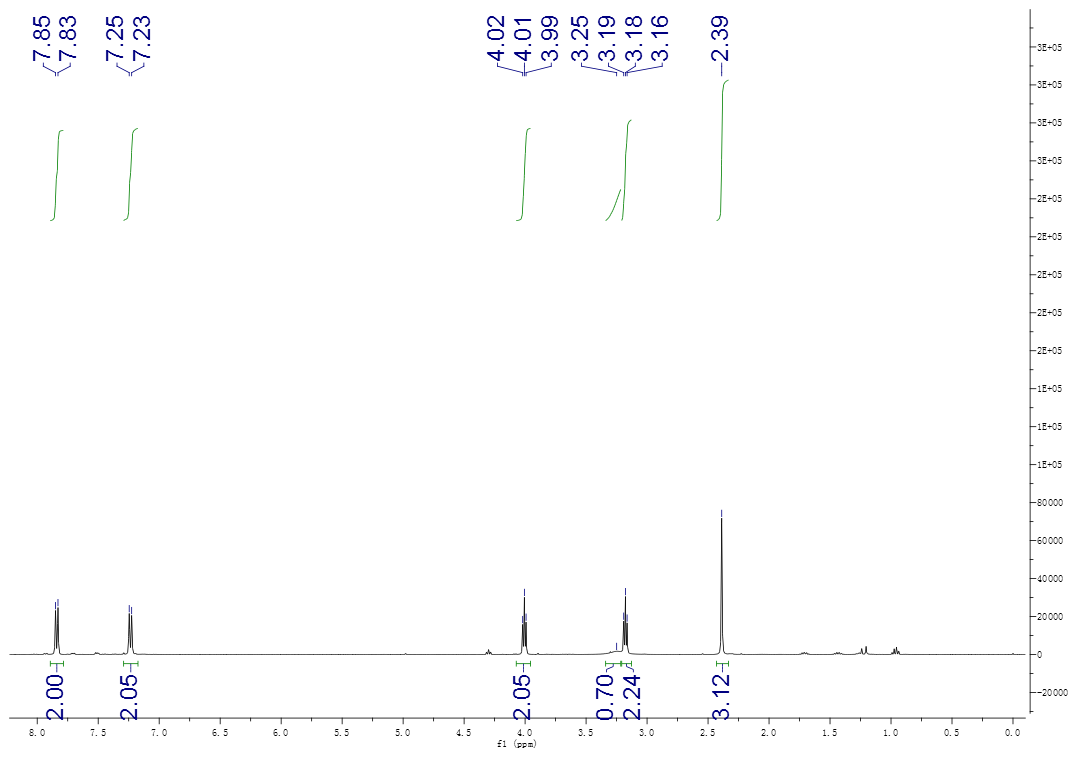


Fig. 17 ^1^H NMR spectrum of product **3i**


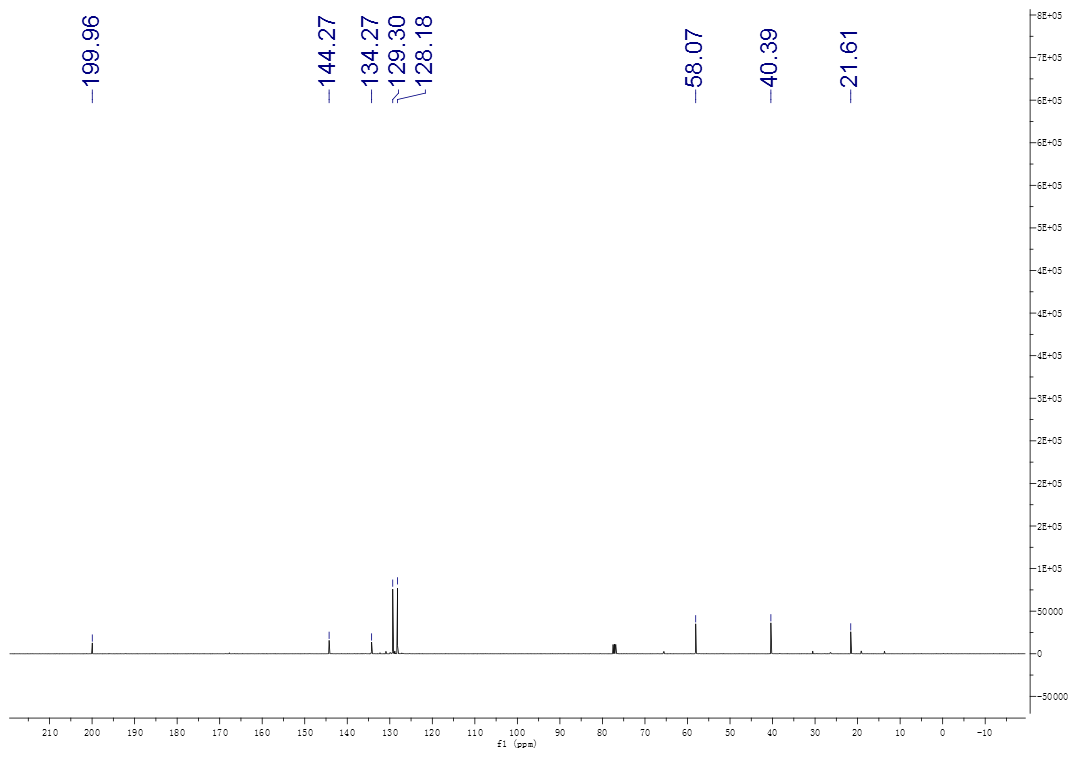


Fig. 18 ^13^C NMR spectrum of product **3i**


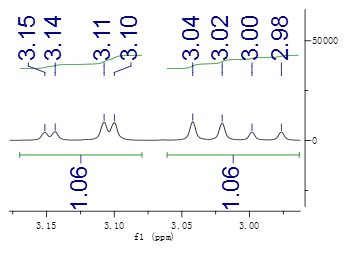

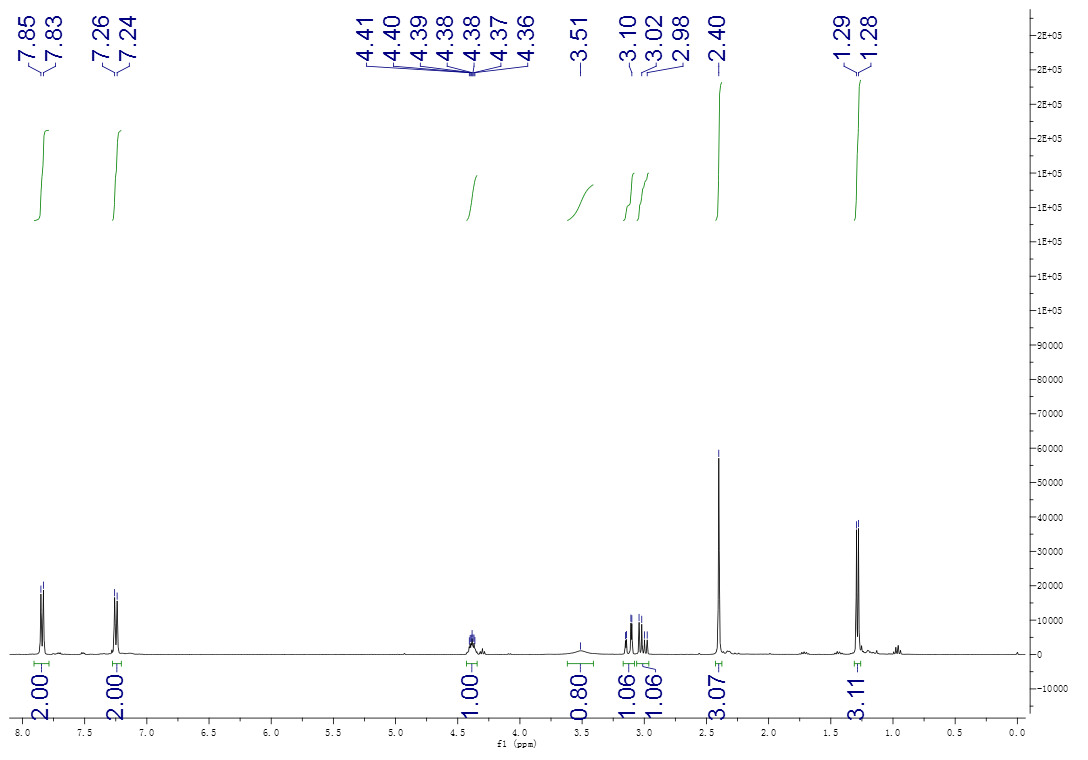


Fig. 19 ^1^H NMR spectrum of product **3j**


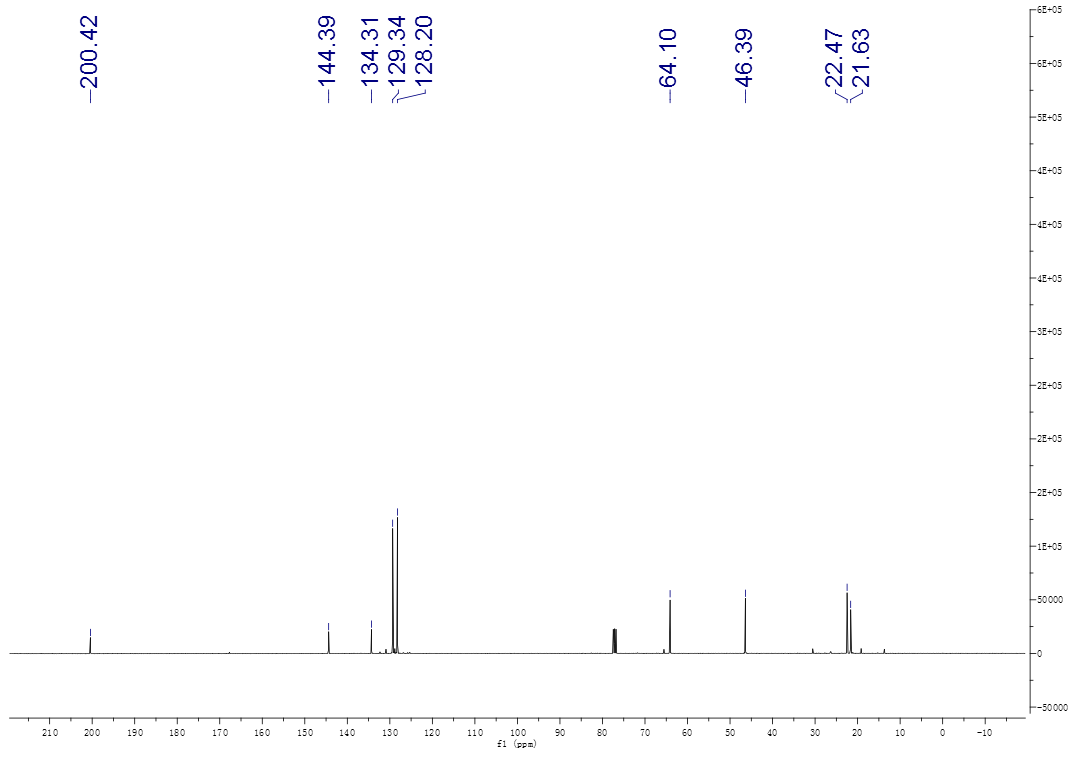


Fig. 20 ^13^C NMR spectrum of product **3j**


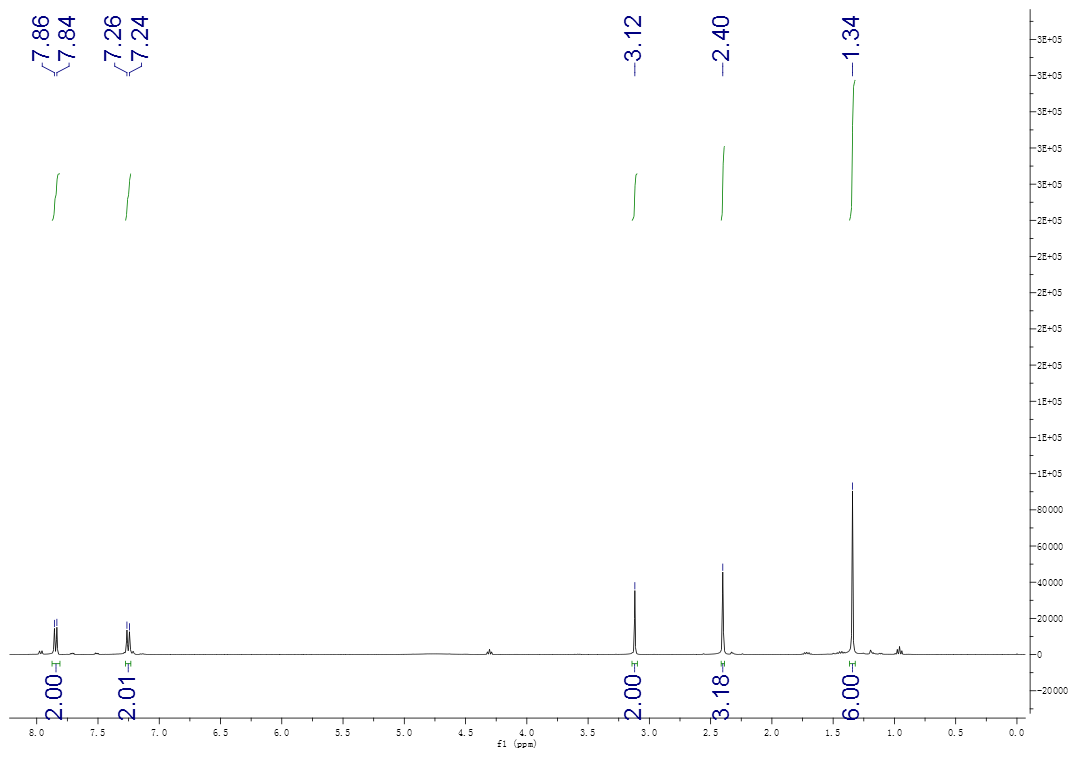


Fig. 21 ^1^H NMR spectrum of product **3k**


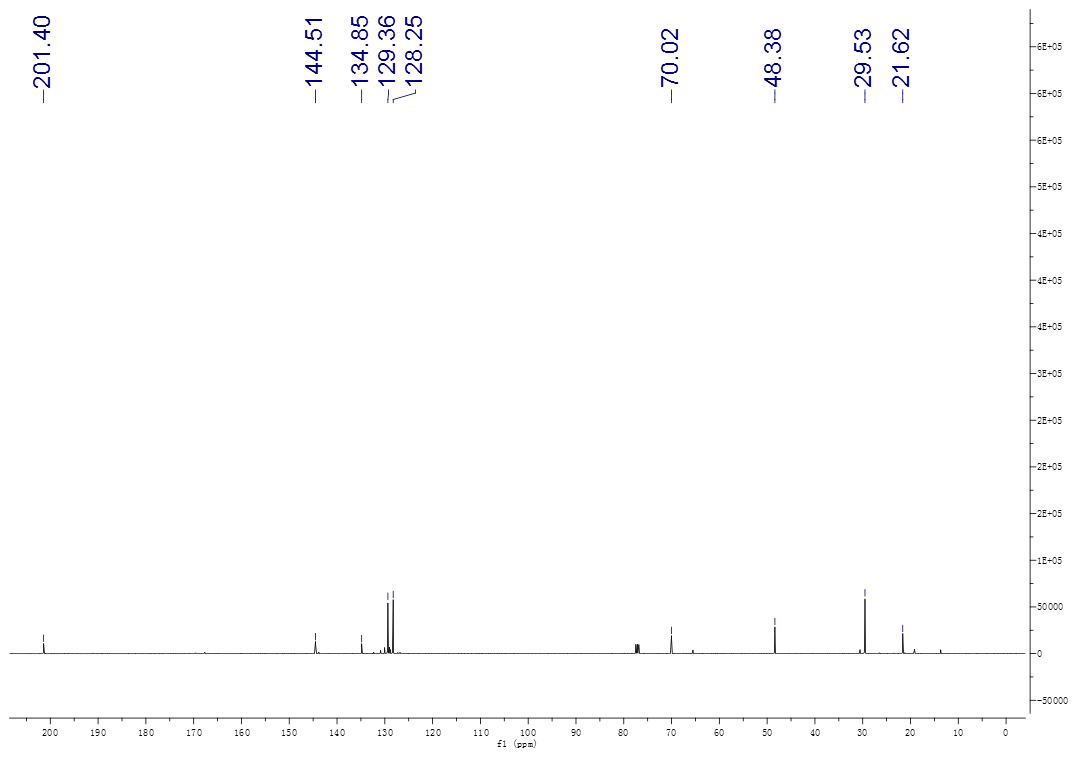


Fig. 22 ^13^C NMR spectrum of product **3k**


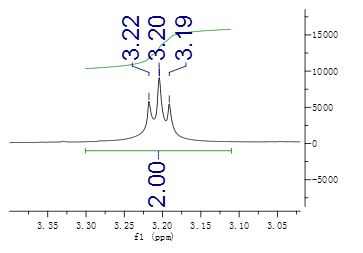

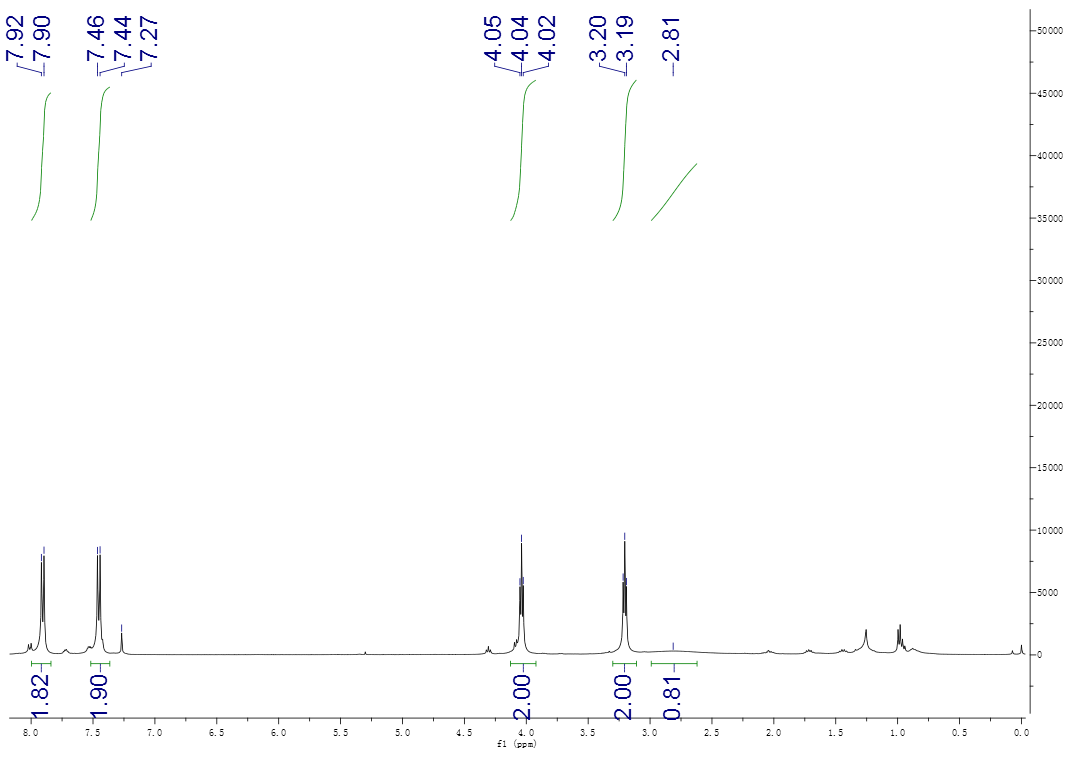


Fig. 23 ^1^H NMR spectrum of product **3l**


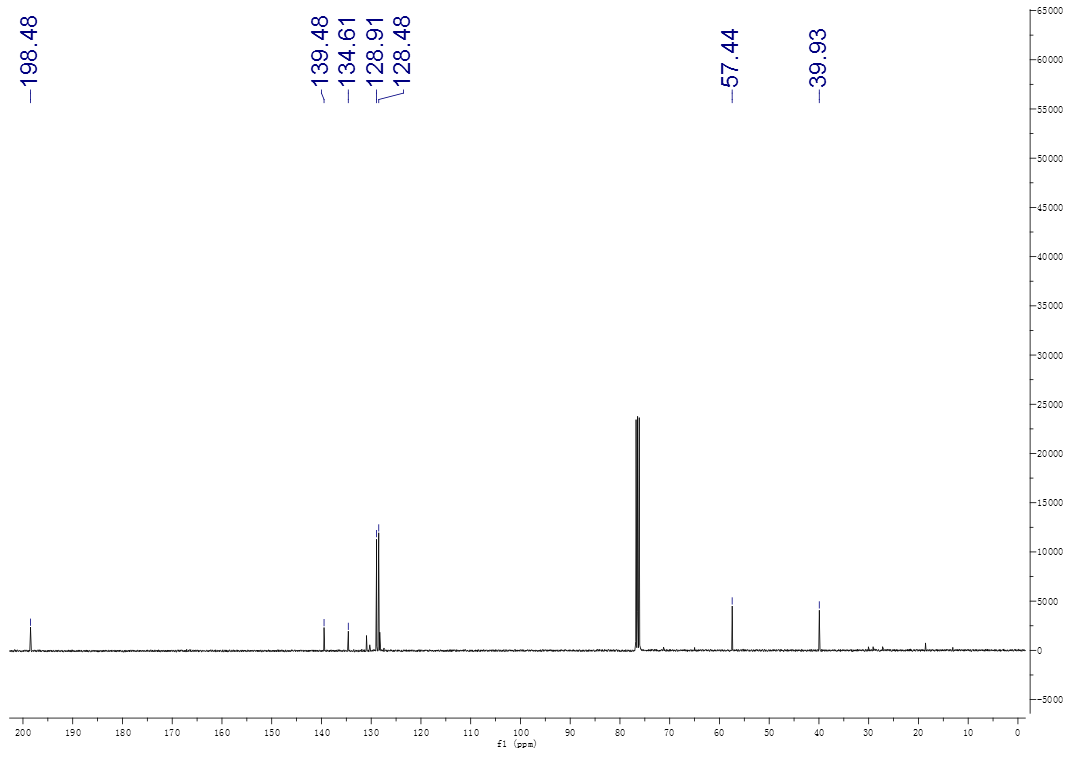


Fig. 24 ^13^C NMR spectrum of product **3l**


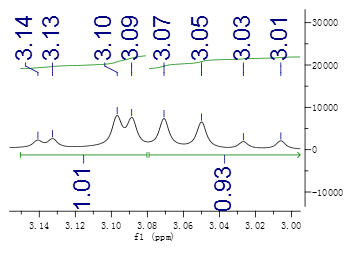

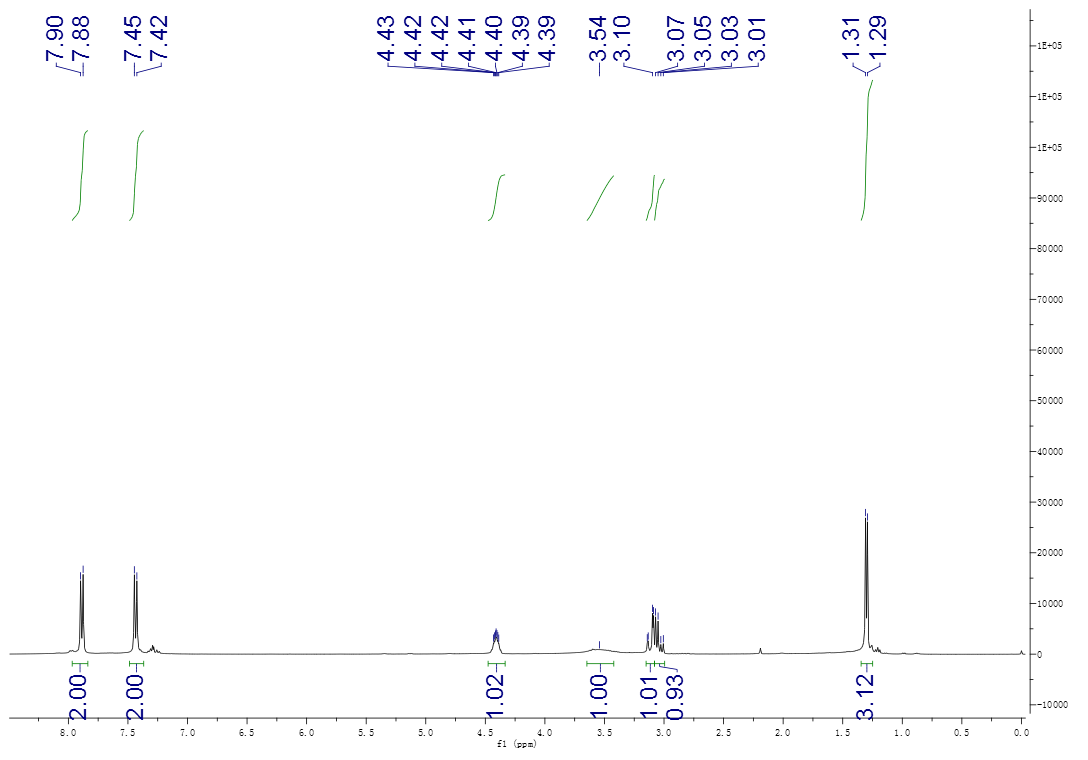


Fig. 25 ^1^H NMR spectrum of product **3m**


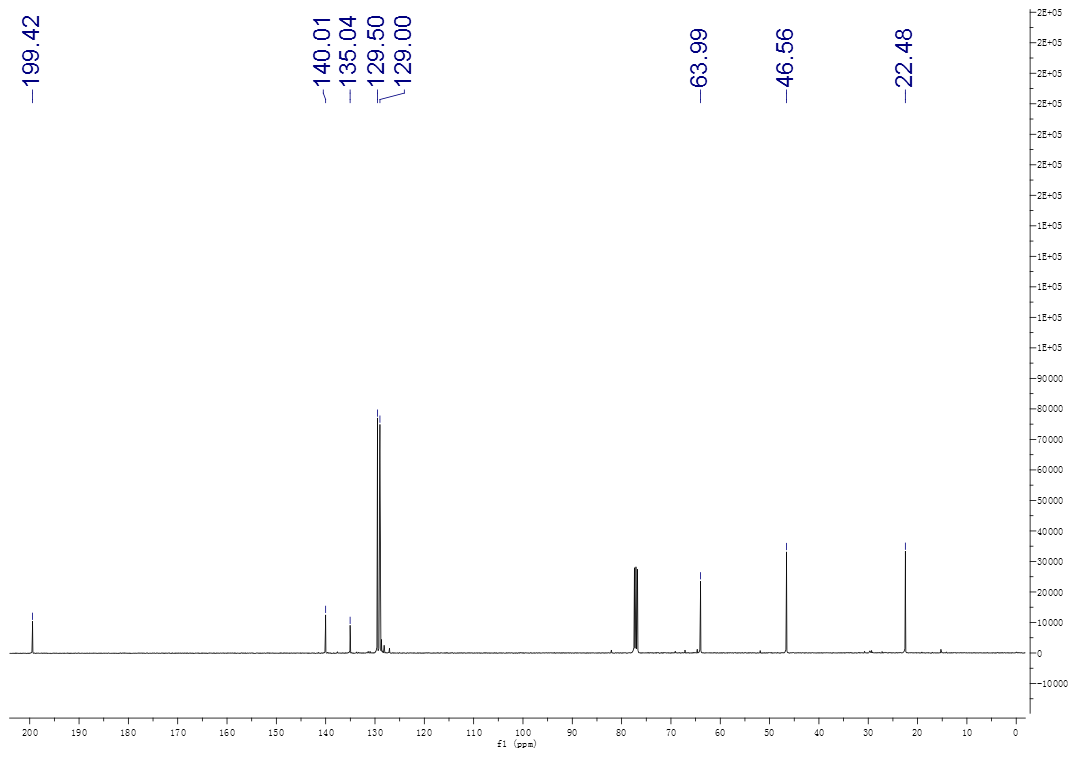


Fig. 26 ^13^C NMR spectrum of product **3m**


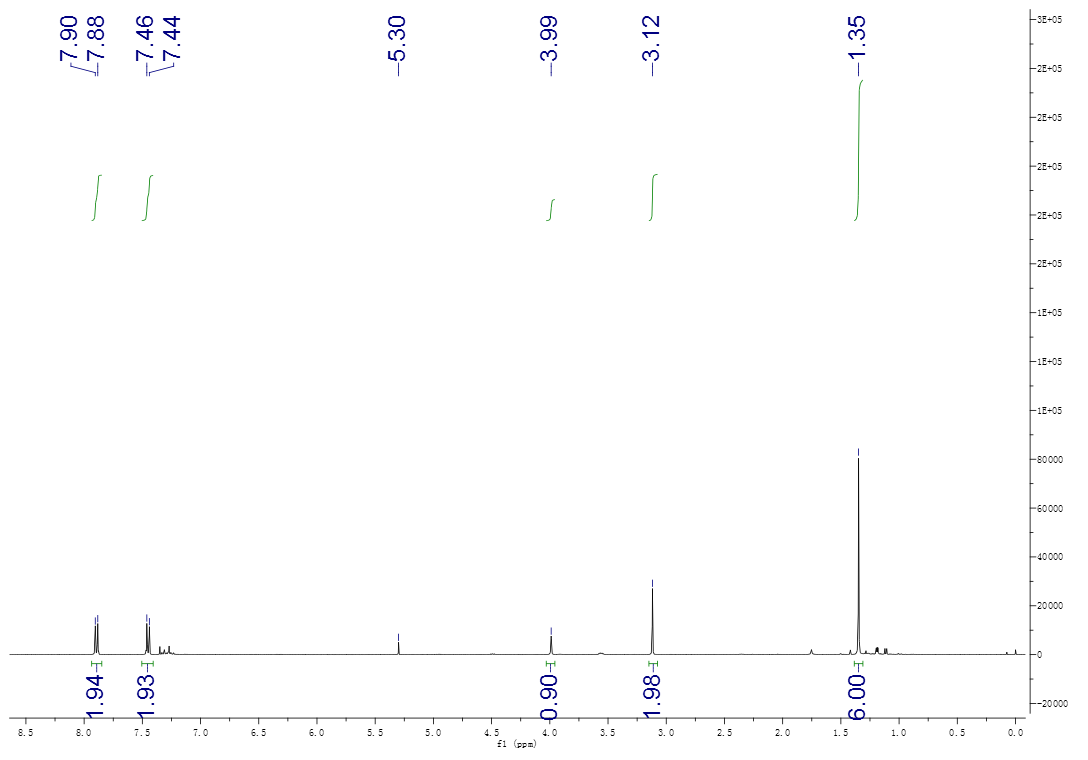


Fig. 27 ^1^H NMR spectrum of product **3n**


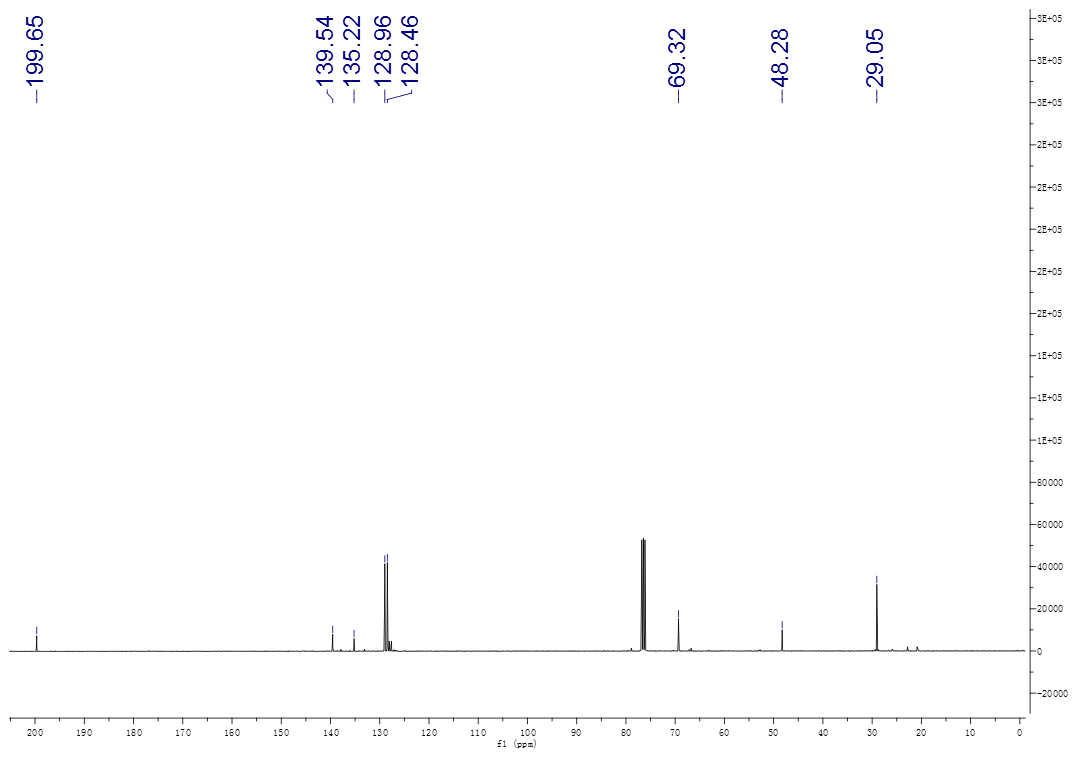


Fig. 28 ^13^C NMR spectrum of product **3n**


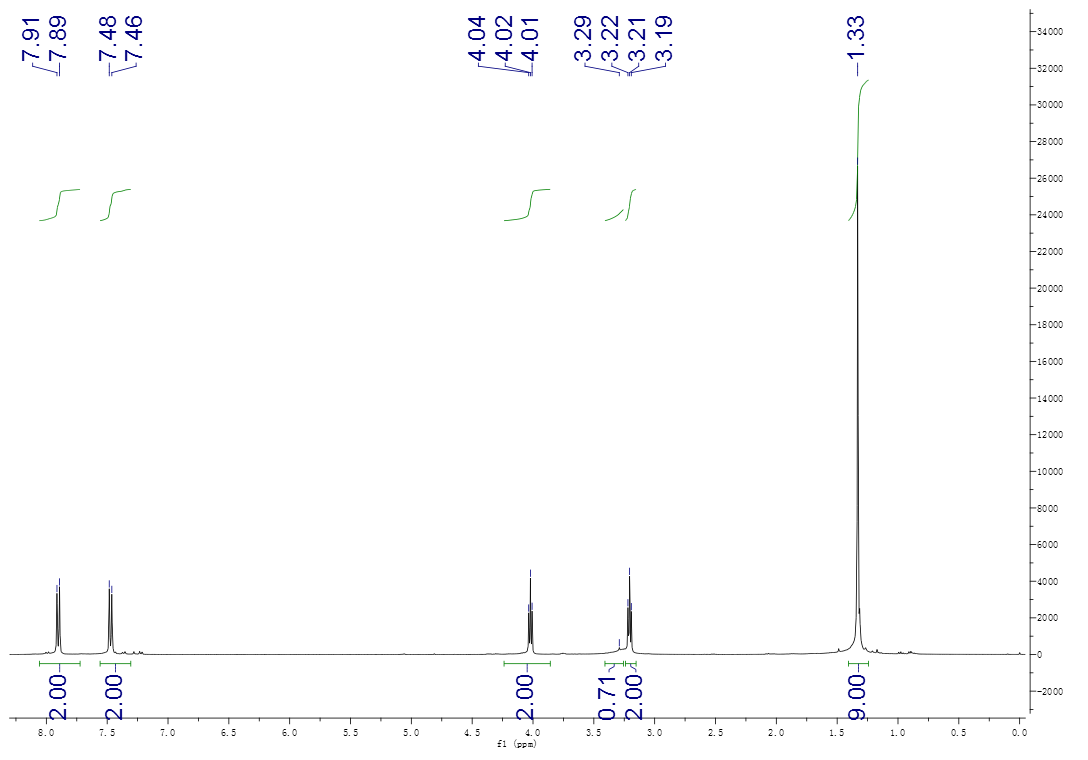


Fig. 29 ^1^H NMR spectrum of product **3o**


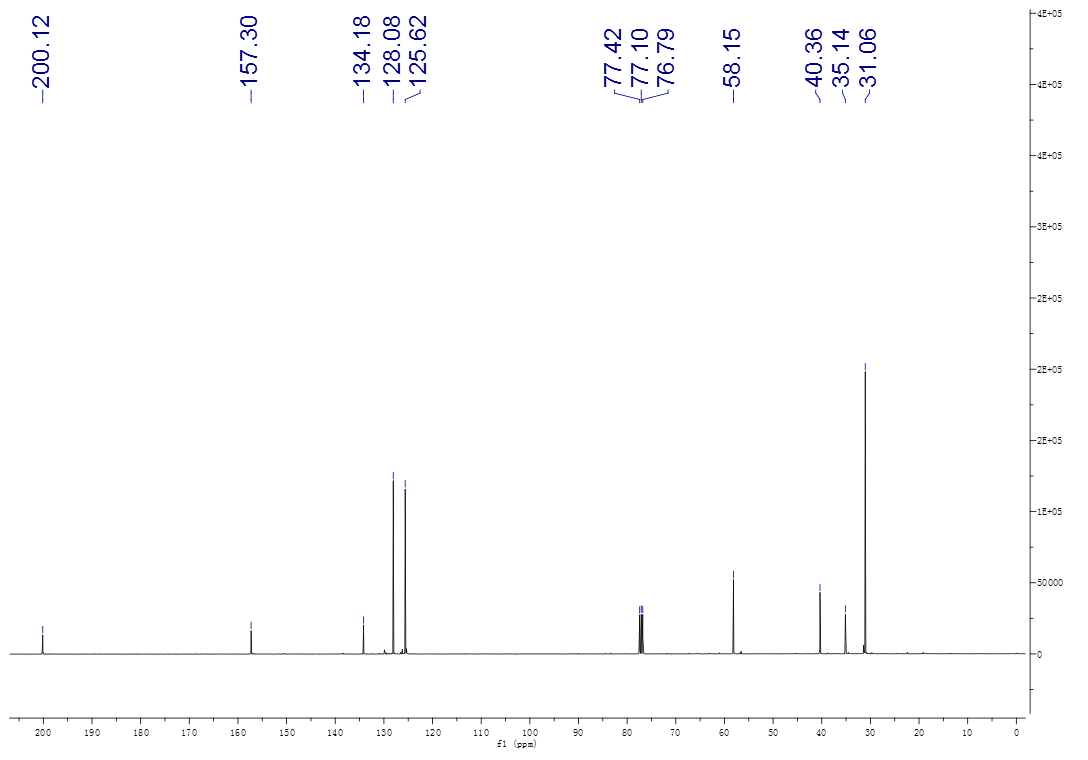


Fig. 30 ^13^C NMR spectrum of product **3o**


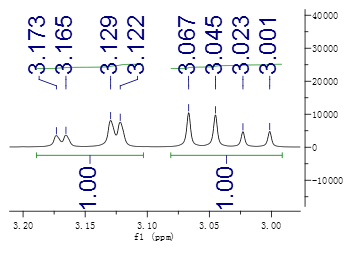

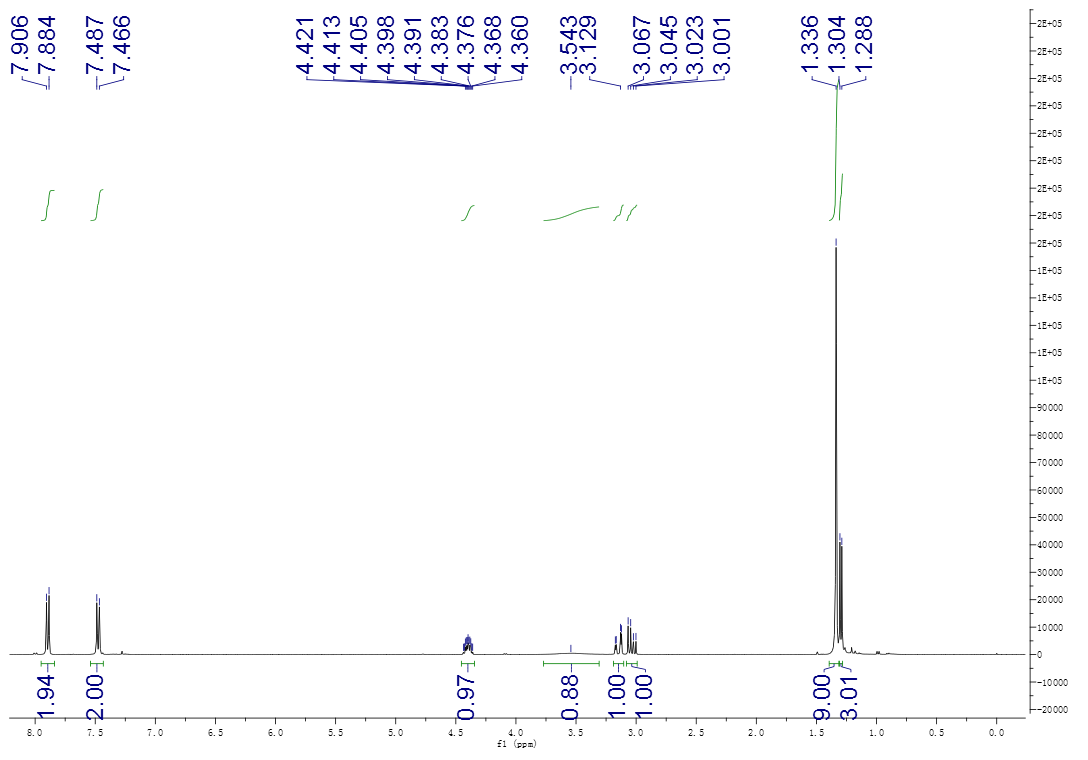


Fig. 31 ^1^H NMR spectrum of product **3p**


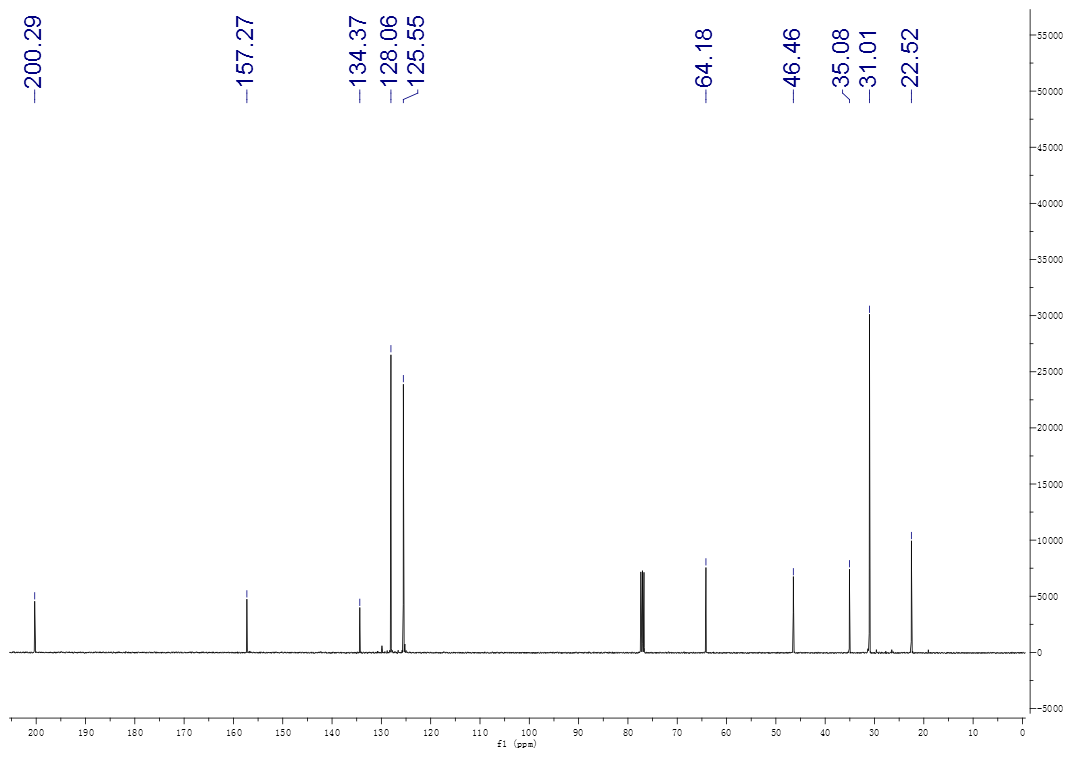


Fig. 32 ^13^C NMR spectrum of product **3p**


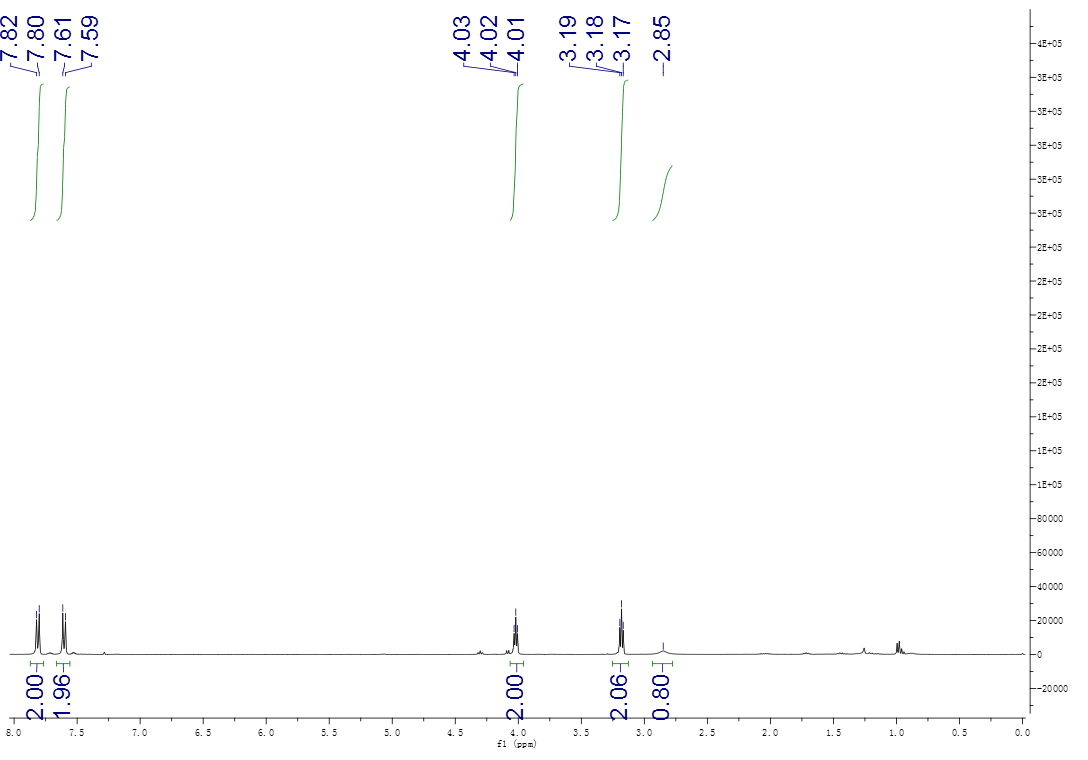


Fig. 33 ^1^H NMR spectrum of product **3q**


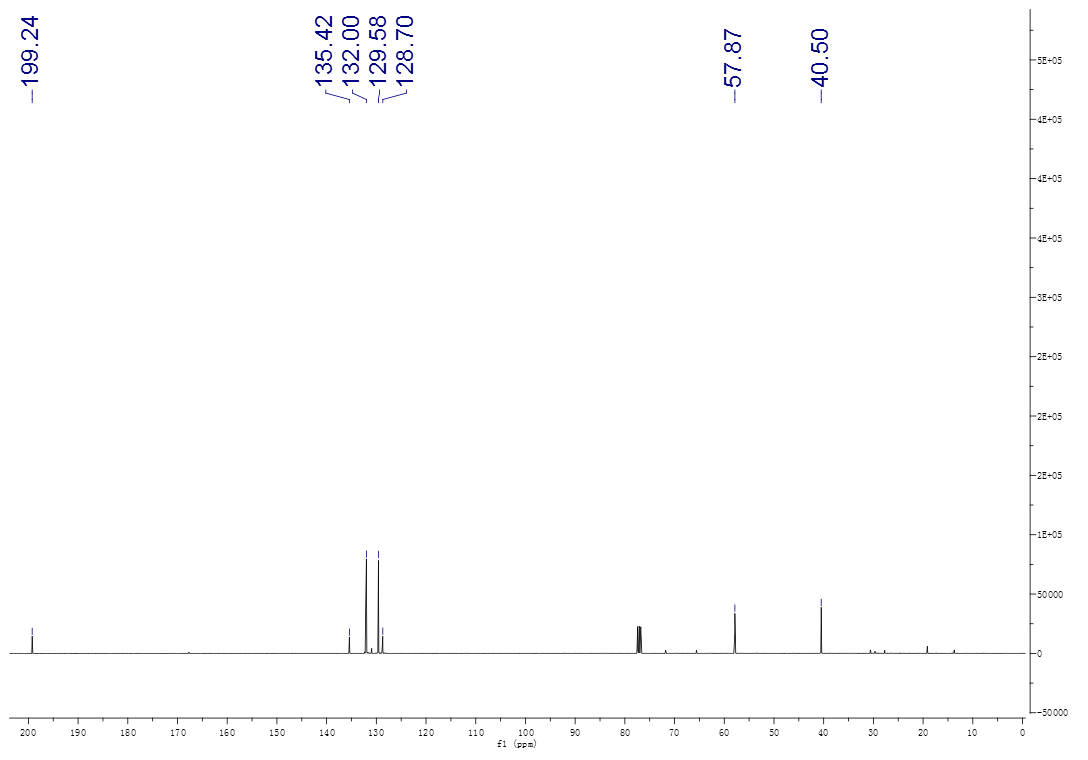


Fig. 34 ^13^C NMR spectrum of product **3q**


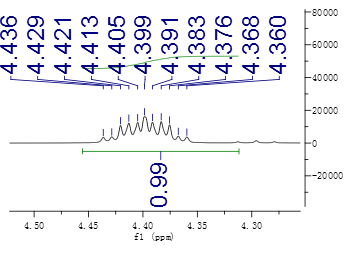

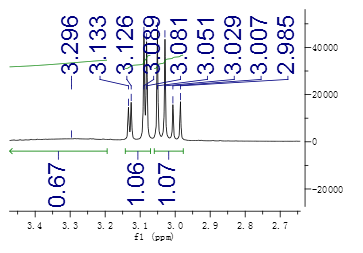

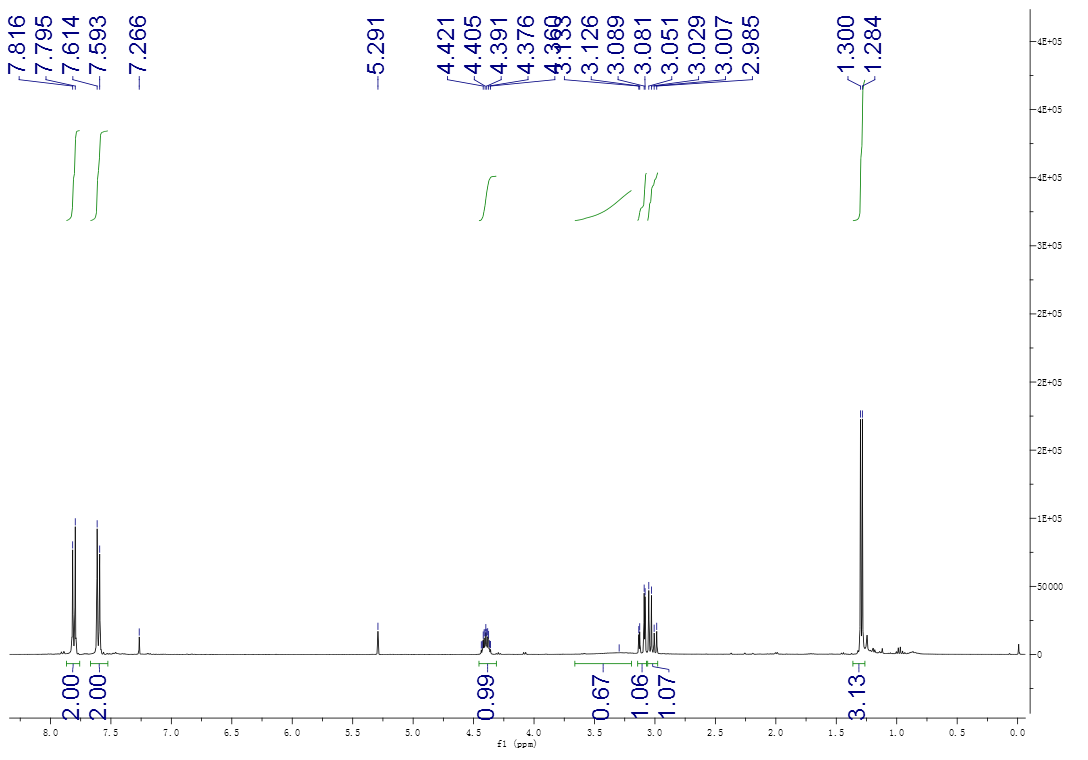


Fig. 35 ^1^H NMR spectrum of product **3r**


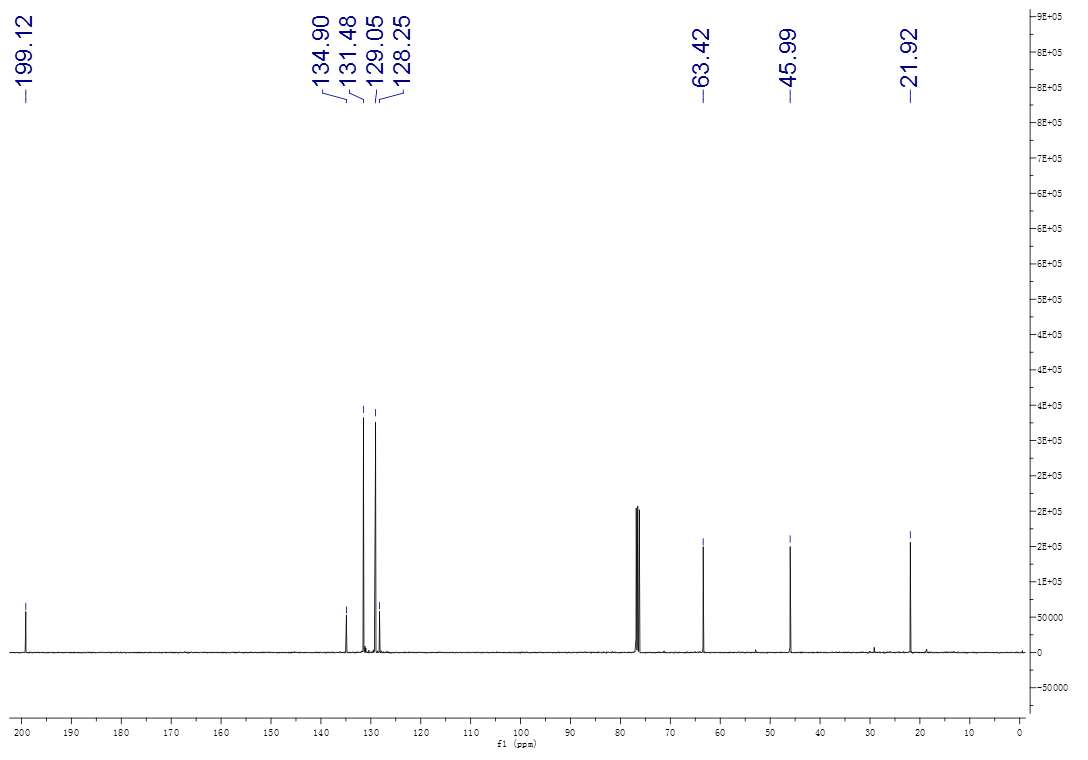


Fig. 36 ^13^C NMR spectrum of product **3r**


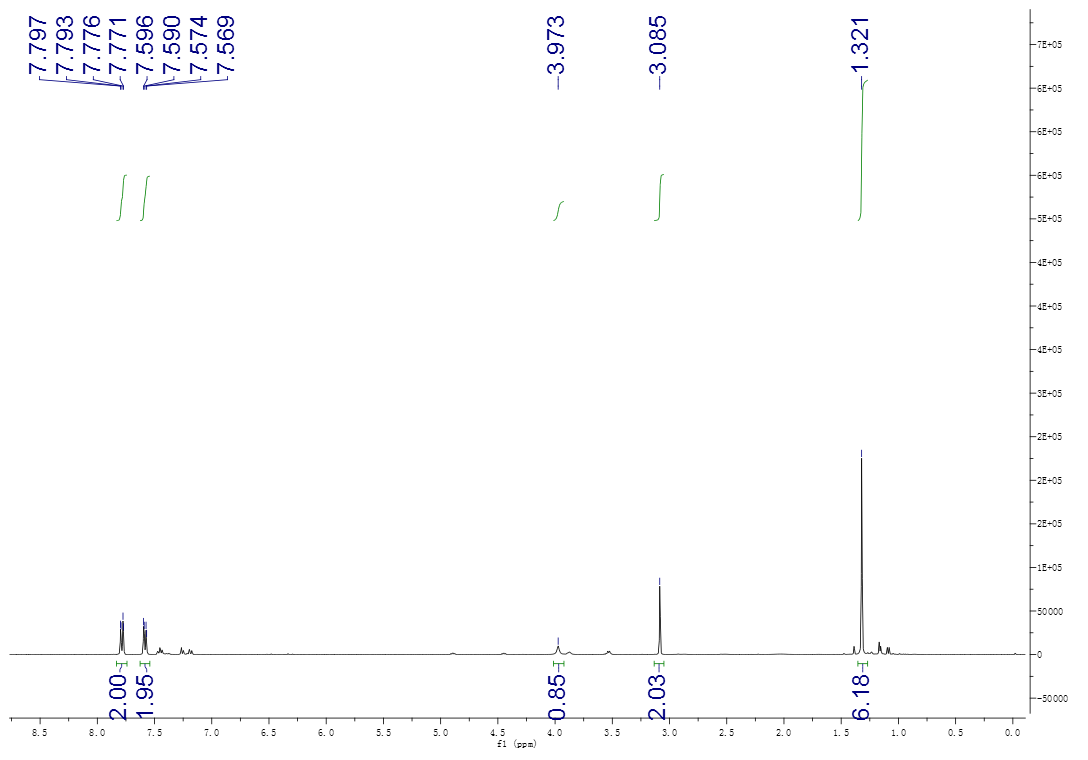


Fig. 37 ^1^H NMR spectrum of product **3s**


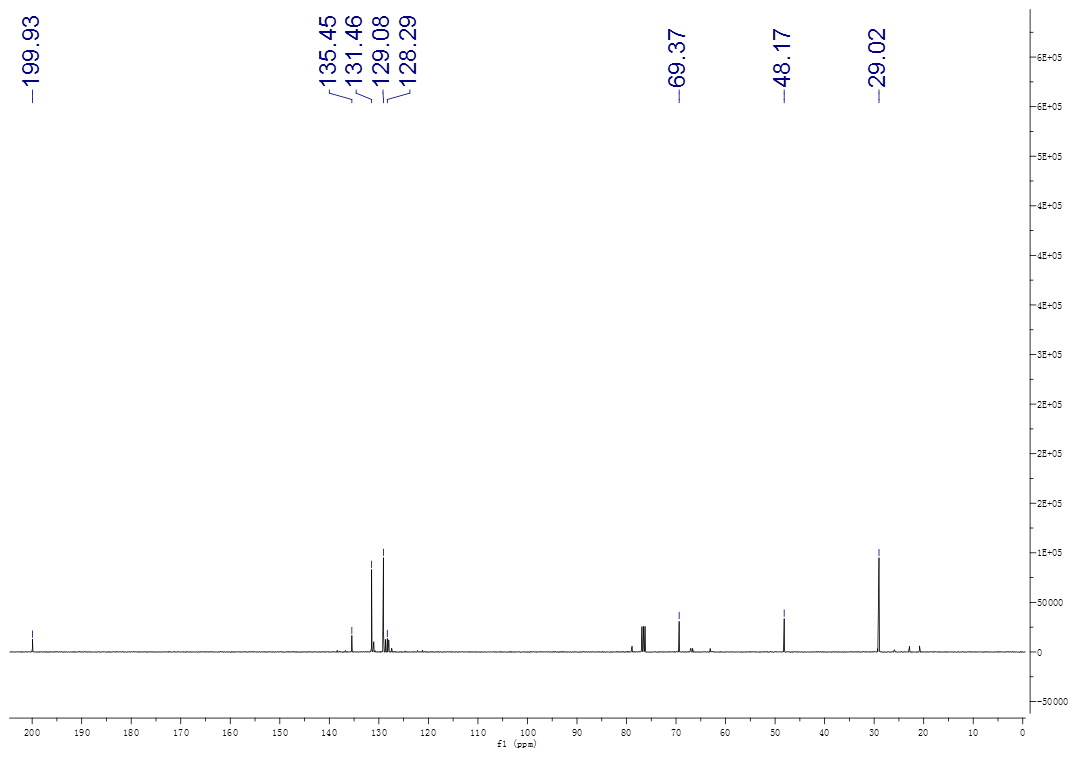


Fig. 38 ^13^C NMR spectrum of product **3s**


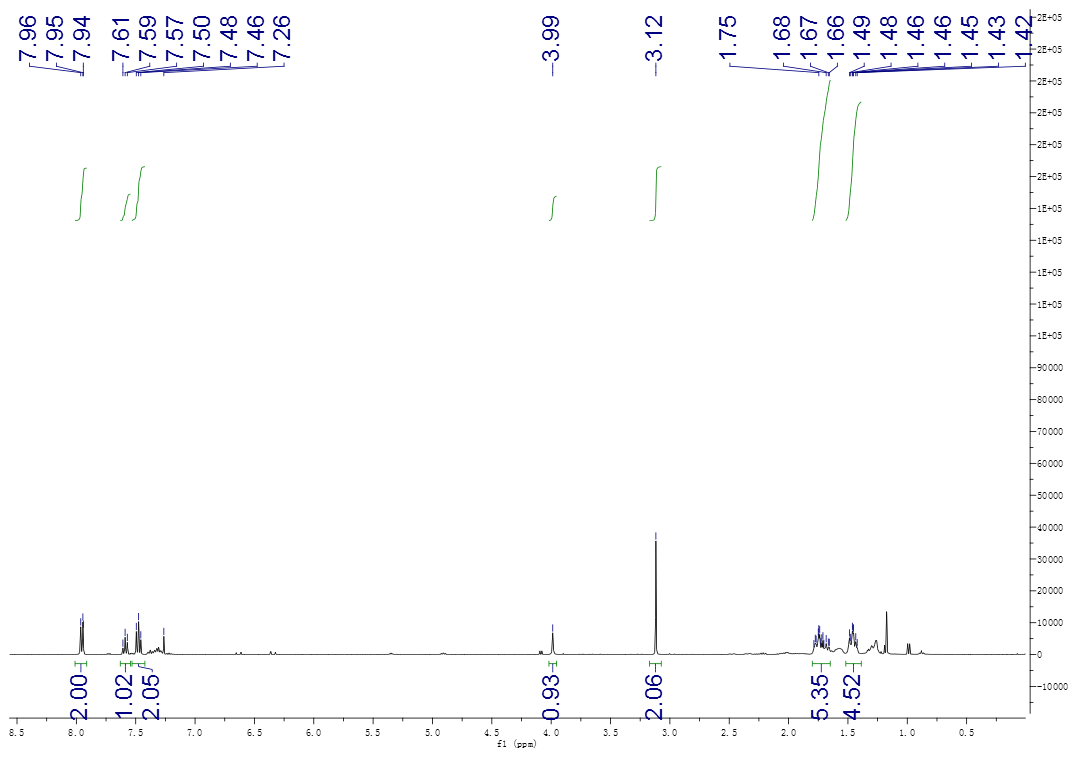


Fig. 39 ^1^H NMR spectrum of product **3t**


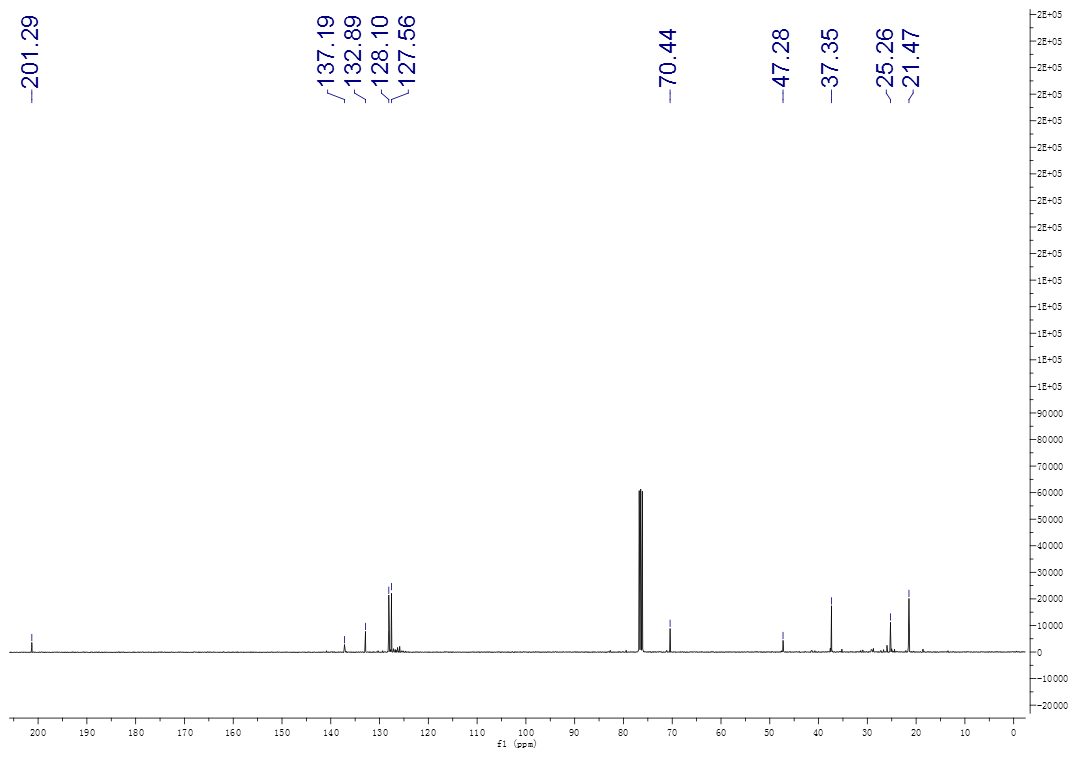


Fig. 40 ^13^C NMR spectrum of product **3t**


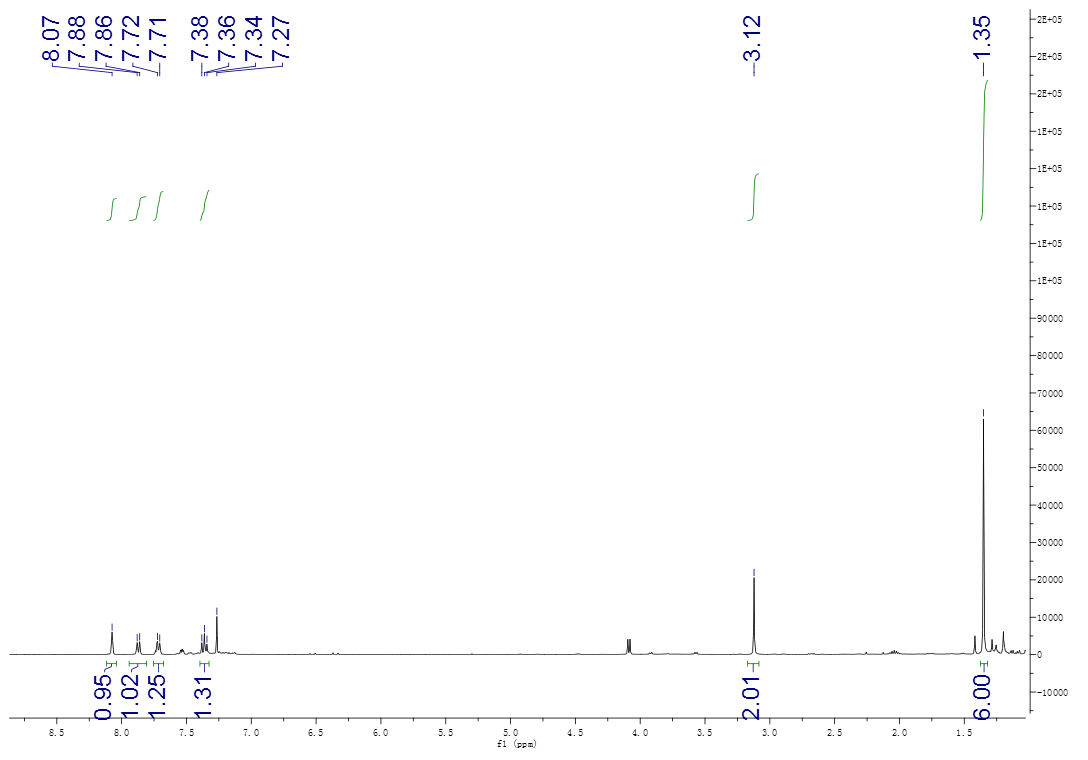


Fig. 41 ^1^H NMR spectrum of product **3u**


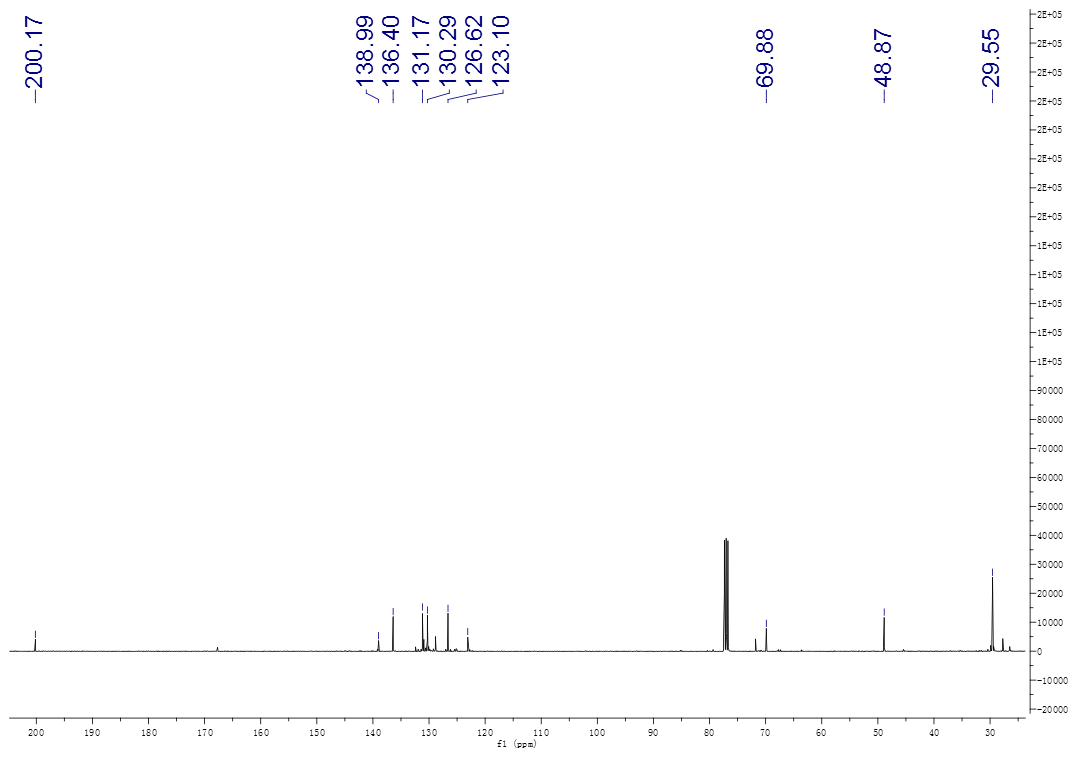


Fig. 42 ^13^C NMR spectrum of product **3u**

1. **HRMS spectra for** **3a** **–** **3u, intermediate C and C’**

Fig. 43 HRMS spectrum of product **3a**

Fig. 44 HRMS spectrum of product **3b**

Fig. 45 HRMS spectrum of product **3c**

Fig. 46 HRMS spectrum of product **3d**

Fig. 47 HRMS spectrum of product **3e**

Fig. 48 HRMS spectrum of product **3f**

Fig. 49 HRMS spectrum of product **3g**

Fig. 50 HRMS spectrum of product **3h**

Fig. 51 HRMS spectrum of product **3i**

Fig. 52 HRMS spectrum of product **3j**

Fig. 53 HRMS spectrum of product **3k**

Fig. 54 HRMS spectrum of product **3l**

Fig. 55 HRMS spectrum of product **3m**

Fig. 56 HRMS spectrum of product **3n**

Fig. 57 HRMS spectrum of product **3o**

Fig. 58 HRMS spectrum of product **3p**

Fig. 59 HRMS spectrum of product **3q**

Fig. 60 HRMS spectrum of product **3r**

Fig. 61 HRMS spectrum of product **3s**

Fig. 62 HRMS spectrum of product **3t**

Fig. 63 HRMS spectrum of product **3u**

Fig. 64 HRMS spectrum of intermediate **C**

Fig. 65 HRMS spectrum of intermediate **C’**
